# Natural Protective Mechanisms of *Cucumis callosus* Leaves in *Escherichia Species*-Induced Urinary Tract Infection: An Integrated In Silico and In Vivo Study

**DOI:** 10.3390/pathogens15010111

**Published:** 2026-01-19

**Authors:** Meenal Sahu, Tripti Paliwal, Radhika Joshi, Arya Kuhu Vishwapriya, Namita Misra, Smita Jain, Gautam Singhvi, Gulshan Kumar, Devesh U. Kapoor, Dipjyoti Chakraborty, Swapnil Sharma

**Affiliations:** 1 Department of Pharmacy, Banasthali Vidyapith, Banasthali 304022, Rajasthan, India; meenalsahu97@gmail.com (M.S.); triptipaliwal43@gmail.com (T.P.); joshiradhika1999@gmail.com (R.J.); kuhuarya2000@gmail.com (A.K.V.); 2 Department of Bioscience & Biotechnology, Banasthali Vidyapith, Banasthali 304022, Rajasthan, India; cdipjyoti@banasthali.in; 3 Department of Physics, Michigan Technological University, Houghton, MI 49931-1295, USA; namitamis@gmail.com; 4 School of Pharmacy & Technology Management, Shri Vile Parle Kelavani Mandal’s Narsee Monjee Institute of Management Studies, Shirpur Campus, Mumbai 425405, Maharashtra, India; smitajain1994@gmail.com; 5 Department of Pharmacy, Birla Institute of Technology and Science (BITS), Pilani 333031, Rajasthan, India; gautam.singhvi@pilani.bits-pilani.ac.in; 6 Department of Chemistry, Banasthali Vidyapith, Banasthali 304022, Rajasthan, India; dr.gulshan.kmr@gmail.com; 7 Department of Pharmaceutics, Dr. Dayaram Patel Pharmacy College, Bardoli 394601, Gujarat, India

**Keywords:** *Cucumis callosus*, urinary tract infection, ayurvedic medicine, bioassay-guided fractionation, LC-MS analysis

## Abstract

Leaves of *Cucumis callosus*, traditionally employed in Ayurvedic medicine for the treatment of urinary disorders, were investigated in depth for their therapeutic potential against bacterially induced urinary tract infection (UTI) for the first time. The present work is the first to explore the antibacterial activity of *C. callosus* leaf fractions with an integrative in silico, in vitro, and in vivo approach. Through bioassay-guided fractionation, the chloroform fraction (F1) was identified as the most active, exhibiting potent activity against Uropathogenic *Escherichia* spp. species. Liquid chromatography–mass spectrometry (LC-MS) analysis of F1 revealed the presence of bioactive compounds, including stigmasterol, 1,2,3,4-tetrahydroisoquinoline, lactose, hydroxy(mesityl)acetic acid, and 2,4-di-tert-butylphenol. Molecular docking studies validated the strong binding affinities of these compounds for bacterial resistance enzymes, including AmpC β-lactamase and carbapenemases, thereby providing plausible mechanisms of antimicrobial action. In vivo studies carried out on female rats infected with *Escherichia* spp. species revealed a dose-dependent reduction in bacterial load, with a significant decrease in urinary tract inflammation upon F1 administration. Histopathological evaluation confirmed the protective effect, with reduced epithelial damage and inflammation in bladder tissues. These findings indicate significant antibacterial and tissue-protective effects of the *C. callosus* leaf fraction F1, supporting its ethnomedicinal use and establishing it as a promising phytotherapeutic agent for the treatment of urinary tract infections.

## 1. Introduction

Urinary tract infection (UTI) is a common microbial infection that occurs when pathogenic microorganisms adhere to, grow in, and colonize certain parts of the host’s urinary tract. UTIs are generally classified into two systems: upper urinary tract infections, which involve the kidneys and ureters, and lower urinary tract infections, which affect the bladder, urethra, and, in females, the vagina [1]. UTI prevalence is disproportionately much more prevalent in females, with risk estimated at around 14 times higher than that seen in males [2]. UTIs infect over 150 million people worldwide annually, with a prevalence rate of 4.3% among women and 1.7% among men [3]. A local study in Jaipur, Rajasthan, India, reported a UTI prevalence of 62.42% among women and 37.67% among men, indicating a substantial gender disparity [4]. Clinically, UTIs present with signs and symptoms such as dysuria (burning or pain during urination), frequency and urgency of urination, suprapubic pain, and cloudy urine. In men, anal pain is possible, whereas in children, infections are usually asymptomatic or nonspecific and present with symptoms like fever, irritability, anorexia, enuresis, or gastrointestinal symptoms like diarrhea [5]. Current treatment regimens are primarily antibiotic regimens, with trimethoprim-sulfamethoxazole, amoxicillin, and nitrofurantoin added, and have been effective for acute infections. Still, long-term antibiotic therapy is typically marked by unwanted side effects such as nausea, vomiting, diarrhea, and headache, and tends to be ineffective in recurrent or chronic urinary tract infections [6]. Antibiotic resistance also makes treatment more challenging and underscores the need for alternative treatment regimens with improved safety profiles. Phytopharmacological constituents have received significant attention due to their possible role in the management of urinary tract infections (UTIs). Ayurvedic medications such as *Vaccinium macrocarpon* (cranberry), *Hydrastis canadensis* (goldenseal), and *Agathosma betulina* (buchu) have been reported to be beneficial in alleviating UTI symptoms and preventing recurrence [7]. In this regard, *Cucumis callosus* (Rottl.) Cong., commonly referred to as ‘Kachri’ or ‘bitter cucumber’, a member of the family Cucurbitaceae, exhibits tremendous therapeutic potential. Indigenous to the arid and semi-arid regions of India, Iran, Nepal, Pakistan, and Myanmar, this plant has traditionally been used in Ayurvedic medicine to treat jaundice, cough, asthma, ulcers, and urinary complaints. Roots and leaves of *C. callosus* have been reported to exhibit a wide range of pharmacological activities, including antioxidant, antibacterial, anticancer, antihyperlipidemic, and antidiabetic activities [8]. *C. callosus* has been used traditionally in Indian and Sri Lankan medicine for an extended period of time. Different plant parts are used in various dosage forms, such as decoctions, powder, paste, and oil forms. Ethnomedicinal surveys reported the use of aqueous preparations, including root decoctions for indigestion and dropsy, water-soaked flower infusions for cardiovascular protection, and fruit pulp for gynecological and rheumatic diseases [9,10]. Seeds and roots are administered orally in powdered form, often with milk, for conditions such as diabetes, fever, and bilious disorders. In contrast, seed oil is traditionally used for neurological disorders and intestinal disturbances, as well as in topical applications. Root and fruit paste is used topically to manage skin diseases, inflammation, and envenomation [11]. Despite its extensive traditional applications, the scientific literature on this plant’s phytochemicals remains limited. However, related Cucumis species have been reported to contain bioactive phytoconstituents, such as cucurbitacins, flavonoids, phenolic compounds, and fatty acids, which may account for their therapeutic properties. Based on these facts, the study hypothesizes that extracts of *C. callosus* contain bioactive phytochemicals responsible for the plant’s reported medicinal properties [12,13,14,15]. The aim of this study was, therefore, to validate the traditional medicinal properties of *C. callosus* through systematic phytochemical and biological studies.

## 2. Materials and Methods

### 2.1. Chemicals and Reagents

The chemicals and reagents were bought from Hi-Media (Maharashtra, India) and Sigma-Aldrich (Burlington, MA, USA). All chemicals were of analytical grade and were used without further purification.

### 2.2. Plant Material

*C. callosus* plant was collected in the months of July–August 2022 from Banasthali Vidyapith, Newai, Tonk, Rajasthan, India. The taxonomist from the Banasthali Vidyapith authenticated it. The voucher specimen number has been deposited in the herbarium as BURI-1726/2023, in Banasthali Vidyapith, Rajasthan, India.

### 2.3. Preparation of Extract and Fractions from Leaves of C. callosus

Hydromethanol in this report refers to a hydroalcoholic solvent composed of 70% methanol and 30% water (*v*/*v*). The shade-dried plant material of *C. callosus* was powdered and divided into three equal portions. Each portion was extracted independently in a single solvent system: methanol, distilled water, or hydro-methanol, to obtain the methanolic (CCM), aqueous (CCAq), and hydro-methanolic (CCHM) extracts, respectively. Each extraction was performed over six days of maceration, with solvent replenishment every 48 h; the solution was composed of the pooled results from all cycles. The resulting extracts were filtered and dried to yield a solid residue, which was then subjected to further biological activity assays. Based on the results of antioxidant and antibacterial activity assays, CCHM exhibited the highest activity and was therefore selected for further fractionation. After drying, CCHM was fractionated using chloroform, n-butanol, and water. These resulted in F1, F2, and F3 fractions, respectively. CCHM is a hydroalcoholic solution; however, the extracts have compounds that are more soluble in the aqueous fraction. Drying and dissolving at specific concentrations were followed for evaluation [16].

### 2.4. Quantitative Estimation of Phytochemical

#### 2.4.1. Total Phenolic Content (TPC)

The total phenolic content of samples was assessed using the Folin–Ciocalteu assay, with gallic acid as a standard. The samples (1 mg/mL) were reacted with Folin–Ciocalteu reagent and sodium carbonate, followed by a 30 min incubation at room temperature, and then absorption at 765 nm. The result was estimated from a standard curve of gallic acid and expressed as mg of gallic acid per gram of extract (mg GAE/g) [17].

#### 2.4.2. Total Flavonoid Content (TFC)

An Aluminum Chloride Colorimetric Assay was performed to determine Total Flavonoid Content. The reference standard used was Quercetin. Stock solutions (1 mg/mL) of the extracts were reacted with AlCl_3_ and potassium acetate, and the mixture was measured at 415 nm after 30 min. The results were quantified in terms of mg of Quercetin per gram extract (mg QE/g) [18].

### 2.5. Liquid Chromatography–Mass Spectrophotometry (LC-MS)

The most active fraction, F1, of the hydromethanolic extract was identified by LC-MS. LC–MS/MS analysis was performed on an Ultimate 3000 UHPLC system coupled to a Thermo Orbitrap Fusion mass spectrometer. Separation was carried outperformed using an Accucore Phenyl-Hexyl column with a gradient of 0.1% formic acid in water (A) and 0.1% formic acid in acetonitrile (B): 0–3 min at 95% A; 18 min at 25% A; 23–30 min at 2% A; 31–35 min to re-equilibrate back to 95% A, at a flow rate of 0.3 mL/min. MS detection was done in positive and negative ESI mode over an *m*/*z* range of 129–1200, with a resolution of 50,000 and a HCD collision energy of 30 eV [19].

### 2.6. Antibacterial Activity

#### 2.6.1. Identification and Characterization of Bacterial Strains

A pathogenic *Escherichia* spp. strain was isolated from drinking water in the vicinity of Banasthali Vidyapith, Rajasthan, India. These bacteria have been characterized as Gram-negative, rod-shaped bacteria. Furthermore, the sequence has been submitted to NCBI BLAST (Basic Local Alignment Search Tool) version 2.17.0+ and GenBank Release 263.0 (accession no. OP649639 is available at https://blast.ncbi.nlm.nih.gov) (22 October 2022).

#### 2.6.2. Antibacterial Susceptibility Activity

The activity of the fraction was assessed by the broth microdilution method, and the minimum bactericidal concentration (MBC) and minimum inhibitory concentration (MIC) were determined according to *Approved Standard, 7th Edition*, NCCLS: Wayne, PA, USA, 2005. The bacteria were grown on nutrient media, prepared by autoclaving at 121 °C for 15–20 min. at 15 psi. For the experiment, 10 mL of nutrient medium was poured into sterile boiling tubes, to which 1 mL of F1 (5–200 µg/mL) and 500 μL of bacterial culture were added. The tubes were then kept in a BOD incubator (Remi, Mumbai, India) at 37 °C for 20–24 h. Later on, the optical density (OD) was analyzed using a UV-Vis spectrophotometer (Labindia, Mumbai, India) at 450 nm. Moreover, 1 mL of the untreated bacterial sample served as the control; similarly, ampicillin served as the standard.

#### 2.6.3. Growth Kinetic Study

This analysis was conducted according to the method of Chouhan and Guleria (2020) with minor modifications [20]. A pure bacterial culture was prepared to approximately 10^8^ CFU/mL using the 0.5 McFarland standard, then treated with the F1 fraction at different concentrations (5–200 μg/mL) and incubated at 37 °C with constant shaking at 120 rpm in an orbital shaker incubator (Remi, Mumbai, India). Bacterial growth after incubation was measured using a UV-Vis spectrophotometer (LabIndia, Mumbai, India) by measuring absorbance at 450 nm at different time intervals from 0 to 48 h. The average OD values of the treated samples and the untreated control were measured at different time intervals and compared. The control was an untreated bacterial culture [21].

#### 2.6.4. Outer Membrane (OM) Permeability Assay

The ability of F1 to induce permeability was measured by the 1-N-phenylnaphthylamine (NPN) uptake assay. During the mid-log phase, bacterial cultures were harvested, washed, and resuspended in a 5 mM HEPES buffer at pH 7.2. Suspension was standardized to an OD of 0.5 ± 0.02 at 450 nm. In a 96-well fluorotiter plate, 100 μL of bacterial suspension, 50 μL of F1 (concentration 5 to 200 μg/mL), and 50 μL of 40 mM NPN were combined. Hydrogen peroxide-treated cells and untreated bacterial cultures served as positive and negative controls, respectively. Fluorescence intensity was measured at 37 °C at each time point until no further increase was observed. Excitation and emission wavelengths were set at 350 nm and 420 nm, respectively, with the assistance of Gen5 v2.06 software on a Synergy H1 microplate reader (BioTek, Winooski, VT, USA) [22].

#### 2.6.5. Inner Membrane (IM) Permeability Assay

F1 has been shown to induce permeabilization of the inner bacterial membrane, as evidenced by the release of cytoplasmic β-galactosidase upon incubation with o-nitrophenyl-β-D-galactopyranoside (ONPG) as the substrate [23]. In a 96-well microplate assay, 90 μL of F1 of different concentrations (5 to 200 μg/mL) was mixed with 10 μL of 30 mM ONPG as per the standard procedure for the determination of outer membrane permeability. The positive control was a bacterial culture treated with 0.5% NaCl, while Triton was used as the negative control. Disruption of the inner membrane was indicated by the formation of o-nitrophenol, which was quantified by measuring absorbance at 420 nm at multiple time points using a microplate reader at 37 °C.

#### 2.6.6. FE-SEM (Field Emission Scanning Electron Microscopy)

The morphological features of the bacteria were examined using FE-SEM to assess the inhibitory activity of F1 against pathogenic bacteria before and after treatment. The non-treated bacterial cells were termed as controls. The treated and untreated bacterial samples were incubated at 37 °C for four hours. Samples were collected during the mid-log phase of bacterial growth and centrifuged at 10,000× *g* rpm for 5 min. The pallet was washed in freshly prepared 50 mM PBS (pH 7.2) and 2.5% glutaraldehyde for 30 min, then rinsed in PBS. The sample was dehydrated using ethanol solutions at concentrations of 30%, 50%, 70%, 90%, and 100%. Subsequently, the desiccated cells were placed in a vacuum oven at 37 °C. Samples were prepared on a sample stage with carbon tape and then coated with palladium ions for 2 min using a plasma sputter-coater (Quorum, Lewes, DE, USA). After which, morphological examinations were made using a FE-SEM (TESCAN Mira 3, Brno, Czech Republic, Germany) at a voltage of 5 kV.

#### 2.6.7. Dehydrogenases Enzymatic Assay

The inhibitory activity of F1 against bacterial dehydrogenase was measured using the modified MTT reduction assay: 3-(4,5-dimethylthiazol-2-yl)-2,5-diphenyltetrazolium bromide [24]. Positively ionized MTT penetrates living cells and interacts with mitochondrial dehydrogenases, oxidizing the bright yellow MTT to the purple formazan. The visual enzymatic conversion was measured at 570 nm. First, 200 μL of culture was added to 10 mL of nutrient medium containing various concentrations (5–200 μg/mL) of F1, adjusted to a 0.5 McFarland turbidity (108 CFU/mL), and incubated at 37 °C for 18–20 h. Then, 1 mL of bacterial cells was collected at 12,000 rpm for 2–3 min. The bacterial pellets were rinsed twice with PBS (pH 7.0) and suspended in the same buffer. To each tube, 0.1 mg/mL MTT reagent was added, and the tubes were incubated in the dark at 37 °C. The negative control was boiled bacterial cells, and the positive control was unboiled bacterial cells. After incubation for various time intervals, the reaction was quenched by the addition of 30 μL of formaldehyde. The intensity of the purple color developed was measured at 570 nm in a UV-Vis spectrophotometer (Labindia, India).

#### 2.6.8. Protein Leakage Assay

F1 was assessed for its ability to leak bacterial protein using the Bradford assay, as described by [25]. The bacterial solution in late log phase was centrifuged for 15 min at 12,000× *g* rpm. Subsequently, bacterial pellets were rinsed twice with 0.85% NaCl buffer. The solution was washed and resuspended in the same buffer. Microbial cells were processed with F1 at various concentrations (5–200 μg/mL) and incubated at 37 °C. Test materials were collected at multiple incubation periods and centrifuged for 3 min at 10,000× *g* rpm. The supernatant protein concentration was measured at OD 275 nm using a UV-Vis spectrophotometer Labindia, India.

### 2.7. In Silico Studies

#### 2.7.1. Molecular Docking of Identified Bioactive Compounds

##### Protein Preparation

The 3-dimensional crystallographic structures of the AmpC and carbapenemase enzymes of the Gram-negative bacterium *Escherichia* spp. were retrieved from the RCSB PDB: 1iel [26] and 6mey [27], respectively. Protein structure modification tasks include modeling missing loop regions, inserting missing atoms, protonating titrable residues, deleting alternate conformations, adding hydrogen atoms, removing heteroatoms, and standardizing atom names. Protein preparation was performed using the CHARMM force field-based molecular dynamics technique [28]. ChemDraw Professional 15.1 was used to sketch the structures of identified bioactive compounds, which were then saved as mol files.

##### Protein Ligand Docking

The LibDock program (Accelrys Discovery Studio 2.0) was used to analyze the binding conformations of identified bioactive compounds with the AmpC and Carbapenemase enzymes, providing insight into potential interactions [29]. The “Best” confirmation method, which employs a poling algorithm to provide a variety of minimal-energy confirmations, was used. The chemicals were docked into the active sites of selected proteins, and the top 10 poses were analyzed to determine the type of interactions.

#### 2.7.2. Density Functional Theory (DFT)

**Figure 7:**
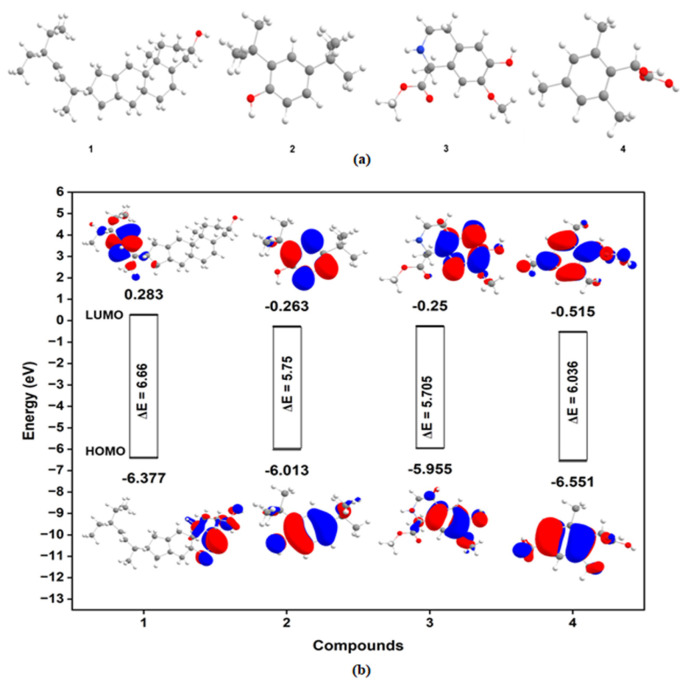
Bioactive compounds (**a**) Optimized structure, (**b**) energy profile of frontier molecular orbitals and topology of orbitals of (1) Stigmasterol, (2) 24-Di-tert-butylphenol, (3) 1234-tetrahydroisoquinolin, and (4) 24-Di-tert-butylphenol.

All bioactive compounds were fully optimized using the Becke three-parameter Lee-Yang-Parr exchange functional (B3LYP) with the 6-311G(d) basis set within the DFT framework. The absence of imaginary frequencies further confirmed the optimized local minima, and the optimized structures are shown in Figure 7a. Further, quantum chemical descriptors (Ionization Potential (I, eV), Energy gap (ΔE; eV), Electronegativity (χ; eV), Chemical Potential (μ; eV), Electron affinity (A), Chemical hardness (η; eV), Electrophilicity index (ω), Chemical Softness (σ)) were calculated from HOMO and LUMO eigen values. All geometry optimization calculations were performed using Gaussian 16. Furthermore, the excitation behavior of the compounds was examined at the TDDFT/B3LYP/6-311++g(d,p) level of theory. The Frontier Molecular Orbital (FMO) theory is employed to predict the molecule’s interaction and reactivity patterns. The energy gap (ΔE) is key to understanding chemical properties, as it determines the stability and reactivity of complexes [30]. The HOMO and LUMO energy was employed to derive a set of physicochemical parameters. HOMO and LUMO of compounds have been investigated and reported in Figure 7b.

### 2.8. Experimental Animals

The study used albino Wistar rats (weighing 150–200 g, female). The study followed CPCSEA guidelines (BV/IAEC/2022/101) and was approved by the Institutional Animal Ethical Committee (IAEC). The animals were kept in polyacrylic cages and under standard conditions, i.e., 60–65% humidity, 25 ± 2 °C temperature, and 12-h light-dark cycle. They were provided with free access to water and dry pellets during the study.

#### 2.8.1. In Vivo Studies

##### Acute Toxicity Study

OCED guideline No. 423 (Annexure 2a) was followed to evaluate the acute toxicity of F1 using female albino Wistar rats. After administration of different doses of F1, animals were monitored for various signs of toxicity and mortality.

##### Bacteria-Induced Chronic Urinary Tract Infection Model

Antibacterial activity of F1 was assessed in female albino rats by the method of [31] with slight modifications. In this model, the bacterial suspension was administered orally to rats at a dose of 200 μL, and infection was confirmed by WBC count using a hematology analyser. Water and food were provided free to the rats. They were divided into six groups of five female rats each. Group I was kept as the normal control. UTI was induced with 200 μL of the isolated bacterial solution in Group II. Groups III and IV were given the herbal standard (cranberry extract, 200 mg/kg) and the antibiotic standard (nitrofurantoin, 50 mg/kg), respectively. Group IV received a low dose of F1 (50 mg/kg) and Group VI a high dose (100 mg/kg) of F1, both administered orally for 14 days.

##### Cytokine Profiling by ELISA

The levels of inflammatory cytokines IL-6 and IL-8 were then determined using an enzyme-linked immunosorbent assay (ELISA) by the method of [32] with certain modifications. Following the manufacturer’s instructions in the Sigma-Aldrich manual, a standard curve was first prepared to enable precise measurement. The samples were then diluted as required before being added to the ELISA plate. The plate was then incubated, washed using a cleaning solution, and dried using filter paper. The antibody was added, washed, and dried before the enzyme conjugate was added. A substrate solution was then added to initiate the colorimetric reaction. The absorbance values were read and standard curves prepared to determine cytokine concentrations.

##### Gene Expression Analysis by Real-Time PCR

Real-time PCR was used to assess mRNA expression of Toll-like receptor 2 (TLR-2) and TLR-4 in bladder tissue, following the procedure of [33] with minor modifications. Bladder tissue was harvested and homogenized. A NanoDrop 2000/2000c spectrophotometer (Thermo Fisher Scientific, Waltham, MA, USA) was used to determine the total RNA concentration by measuring absorbance at 260 and 280 nm. All the samples showed readings between 1.8 and 2.0. cDNA synthesis was performedperformed using Verso cDNA synthesis kit (Thermo Fisher Scientific, USA). RT-PCR of TLR-2 and TLR-4 genes (Table 5) was done using the Bio-Rad CFX96™ RT-PCR System (Bio-Rad, Hercules, CA, USA), and the 2^−ΔΔCt method was utilized to determine the levels of gene expression. Each reaction was provided with a 20 μL mixture comprising 1 μL cDNA, 2 μL primers, and 10 μL SYBR Green PCR Master Mix. The amplification conditions were pre-denaturation at 95 °C for 30 s, denaturation at 95 °C for 5 s, annealing at 60 °C for 34 s, and extension at 60 °C for 1 min. Forty amplification cycles were executed. The annealing extension condition of the last amplification cycle was 72 °C for 10 min.

##### Biochemical Studies

Bladder tissue was homogenized in 10 mL of PBS at 4 °C, and then centrifuged at 12,000× *g* rpm for 30 min. The supernatant obtained was collected, aliquoted, and used for protein and enzymatic assays. Protein content in bladder tissue was estimated by the Bradford method, using bovine serum albumin as the standard. Lipid peroxidation was assessed by measuring TBARS concentration in tissues using a previously described method. The tissue homogenates were also used to estimate catalase and reduced glutathione (GSH) activities [34].

##### Histopathology

Histopathological analysis of the bladder tissue was performed on 5-µm paraffin-embedded tissue sections stained with hematoxylin and eosin. The images were captured using MICAPS 3.1 software with a LABOMED trinocular phase-contrast microscope equipped with a MICAPS FERLAF050 camera MICAPS, Seoul, Republic of Korea).

## 3. Statistical Analysis

GraphPad Prism 8.0 software was utilized for statistical analysis. The data were analyzed using one- and two-way ANOVA to compare groups. All mean values in each group are signified as mean ± SD (*n* = 5). A *p*-value of below 0.05 was statistically significant.

## 4. Results

### 4.1. Quantitative Estimation of Phytochemical

#### 4.1.1. Total Phenolic Content (TPC)

**Figure 1:**
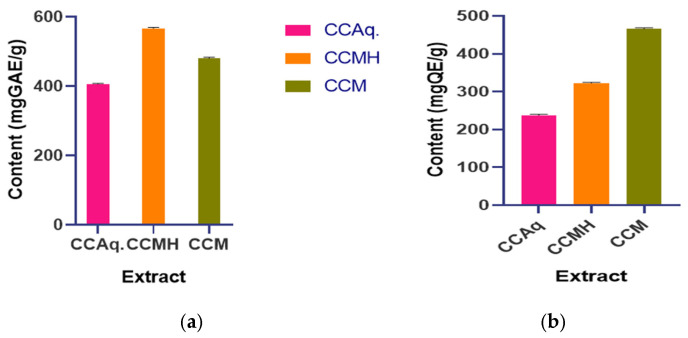
(**a**) TPC and (**b**) TFC of *C. callosus*. The error bar represents the standard error of the mean.

Overall, the TPCs of *C. callosus* extracts differed across the tested solvents. The highest values were obtained from the CCHM at 566.45 mg GAE/g, followed by CCM at 480.43 mg GAE/g and the CCAq at 405.88 mg GAE/g (Figure 1a). The quantification was based on a gallic acid calibration curve, which showed good linearity (R^2^ = 0.993).

#### 4.1.2. Total Flavonoid Content (TFC)

Analysis of TFC revealed a distinct distribution pattern among the extracts. The CCM presented the highest concentration of flavonoids, 466.09 mg QE/g, whereas the hydromethanolic (CCHM; 322.72 mg QE/g) and aqueous extracts (CCAq; 237.42 mg QE/g) had lower concentrations (Figure 1b). TFC values were calculated using a quercetin standard curve, which showed excellent linearity (R^2^ = 0.999).

### 4.2. Antibacterial Activity

The MIC and MBC values of CCAq, CCM, and CCHM were compared for activity against *Escherichia* spp. to identify the most potent extract for further fractionation. Among the three extracts, the CCHM was the most potent, exhibiting the lowest MIC and MBC values.

The CCHM had MIC and MBC values of 5 µg/mL and 300 µg/mL, respectively, and exhibited strong inhibitory and bactericidal activity. On the other hand, the CCM showed moderate activity with MIC (5 µg/mL) and MBC (350 µg/mL) values slightly greater than the aqueous extract. The CCAq demonstrated the lowest activity, with relatively high MIC (5 µg/mL) and MBC (350 µg/mL) values, indicating minimal efficacy against the tested pathogens (Table 1).

Based on these results, the CCHM was selected for further fractionation due to its significant antibacterial activity.

#### Antibacterial Activity of Susceptibility

The MIC and MBC of fractions against the pathogenic *Escherichia* spp. bacteria were therefore determined to test their antibacterial activity. Fraction F1 exhibited the highest antibacterial potency, with a MIC value of just 2 μg/mL, followed by F2 (4 μg/mL) and F3 (20 μg/mL). These MIC values were compared with those of the standard antibiotic ampicillin (5 μg/mL), as presented in Table 2.

Similarly, the MBC results revealed that F1 exhibited the highest bactericidal activity at 195 μg/mL, followed by F2 and F3, both at 200 μg/mL. The MBC values were also compared with ampicillin (200 μg/mL) and are presented in Table 2.

The in vitro antibacterial analysis confirmed that the selected *C. callosus* fraction exhibited increased antibacterial activity against the target strain. Based on these results, the most potent fraction, F1, was chosen for further studies.

### 4.3. LC-MS Analysis of F1

The LC-MS analysis of F1 shown the presence of 21 bioactive compounds (Table 3), (Figures S2–S12), key constituents of the plant include p-Cresol, Stigmasterol, Hydroxy (mesityl) acetic acid, Hexadecanoic acid, Oleic Acid and Octadecanoic acid, etc. Notably, the majority of identified active bioactive compounds belong to the Steroid and phenolic families.

#### 4.3.1. Growth Kinetics Study

**Figure 2:**
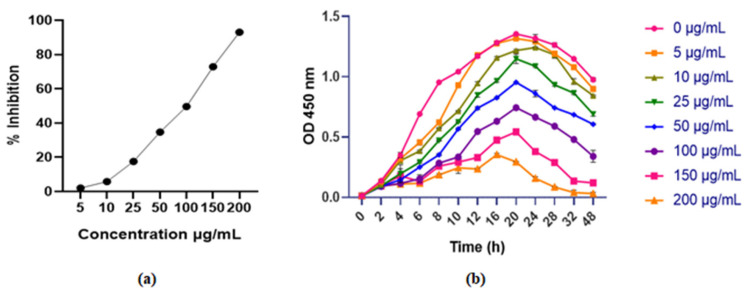
(**a**) F1’s % inhibition, (**b**) growth kinetics study at various F1’s concentrations against an isolated pathogenic *Escherichia* spp. bacteria. The error bar indicates the standard deviation of the mean.

Growth kinetics of pathogenic *Escherichia* spp. were studied with and without F1, and the results are shown in Figure 2b. F1’s treatment significantly inhibited *Escherichia* spp. at concentrations varying from 5–200 μg/mL. Increasing the incubation duration resulted in the complete killing of bacterial cells. The results show that F1 engages with bacterial cell membranes and causes bacterial lysis in a time- and concentration-dependent manner. The study found that F1’s antibacterial activity was influenced by both fraction concentration and treatment period.

#### 4.3.2. Outer-Inner Membrane Permeability Assay

**Figure 3:**
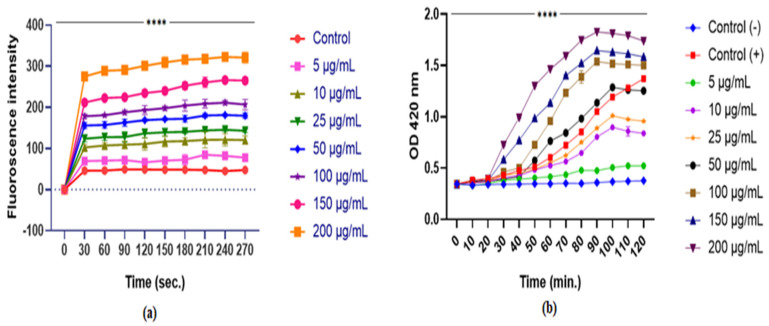
The impact of F1 on the (**a**) permeabilization of the OM, (**b**) cytoplasmic β-galactosidase released from the inner membrane of bacterial cells was measured at different concentrations of F1. Triton and 0.5% NaCl were used as positive and negative controls, respectively. Statistical significance was assessed using a two-way ANOVA and compared with the negative control. Significance is denoted as **** *p* < 0.0001, along with the standard error of the mean.

The capability of F1 to break the OM of *Escherichia* spp. was tested utilizing the NPN assay. NPN is a fluorescent probe that exhibits higher fluorescence in hydrophobic conditions, such as the bacterial membrane, and less fluorescence in aqueous conditions. F1 destroyed bacterial cell membranes in a concentration-dependent manner, as seen by increasing fluorescence intensity [35] (Figure 3a).

The ONPG assay was used to investigate the effect of F1 on inner membrane integrity. Adding graded concentrations of F1 to bacterial media resulted in a significant time-dependent increase in OD at 420 nm. ONPG’s interaction with cytoplasmic galactosidase leads to the formation of ortho-nitrophenol in the medium, causing an increase in absorbance. Leaking cytoplasmic galactosidase requires dissolution of the inner bacterial membrane. The results support F1’s ability to disrupt the inner bacterial membrane (Figure 3b).

#### 4.3.3. Field Emission Scanning Electron Microscopy

**Figure 4:**
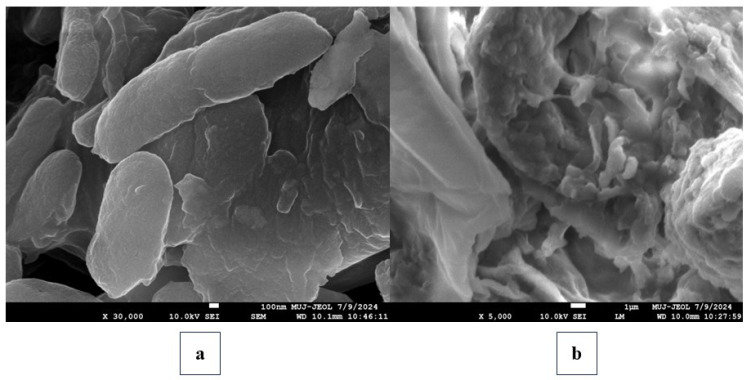
FE-SEM images of (**a**) non-treated bacteria, (**b**) 4-h F1-treated bacterial cells.

FE-SEM analysis was used to assess the morphological alterations in bacterial cells caused by F1 (Figure 4). Untreated bacterial samples had well-defined cell structures and smooth surfaces (Figure 4a). In contrast, F1 treatment caused aggregation and clumping of the bacterial cell membrane, and the cell wall was damaged entirely and ruptured, as shown in Figure 4b. FE-SEM study shows that F1 degrades and ruptures bacterial cell membranes, causing aggregation and clumping in high-resolution images.

#### 4.3.4. Dehydrogenase Enzymatic Assay

**Figure 5:**
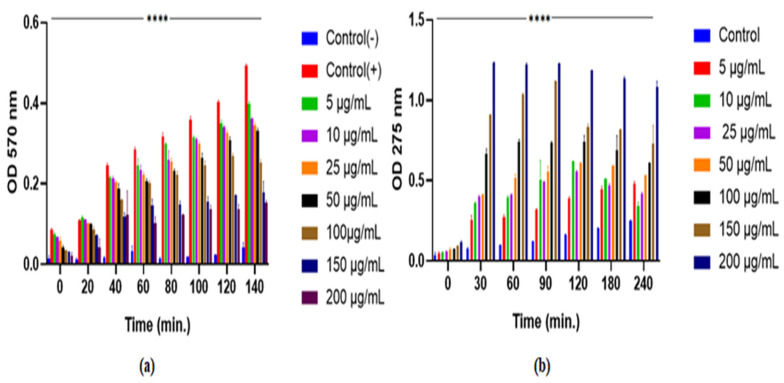
F1-treated bacterial cells (**a**) enzymatic dehydrogenase activity was assayed using boiled and unboiled bacterial cells as negative and positive controls, respectively (**b**) protein leakage was determined. Two-way ANOVA was conducted, and **** *p* < 0.0001 was taken as statistically significant. The error bars presented are the standard error of the mean.

The MTT assay was used to evaluate F1’s ability to inhibit bacterial dehydrogenase. F1 inhibited bacterial dehydrogenase in a time- and concentration-dependent manner relative to the positive control. F1 (200 μg/mL) inhibited bacterial dehydrogenase immediately and reached maximal effectiveness at 100 min, as shown in Figure 5a. F1 exhibits significant bactericidal activity by disintegrating outer and inner membranes and inhibiting bacterial mitochondrial dehydrogenase.

#### 4.3.5. Protein Leakage Assay

F1’s ability to prevent bacterial cell leakage was tested by measuring the OD of bacterial samples over various incubation intervals. F1’s treatment inhibited bacterial cells in a concentration- and time-dependent manner, resulting in higher absorbance and protein leakage than the control (Figure 5b). The leakage of proteins and nucleotides from disrupted bacterial cells was assessed by monitoring OD at 275 nm as a function of exposure duration. It was noted that increasing the F1 concentration and exposure time led to faster cell protein leakage compared with controls, and that F1 induced considerable bacterial lysis and continuous leakage of critical metabolites from bacterial cells [25].

### 4.4. In Silico Studies

#### 4.4.1. Molecular Docking of Identified Bioactive Compounds

**Figure 6:**
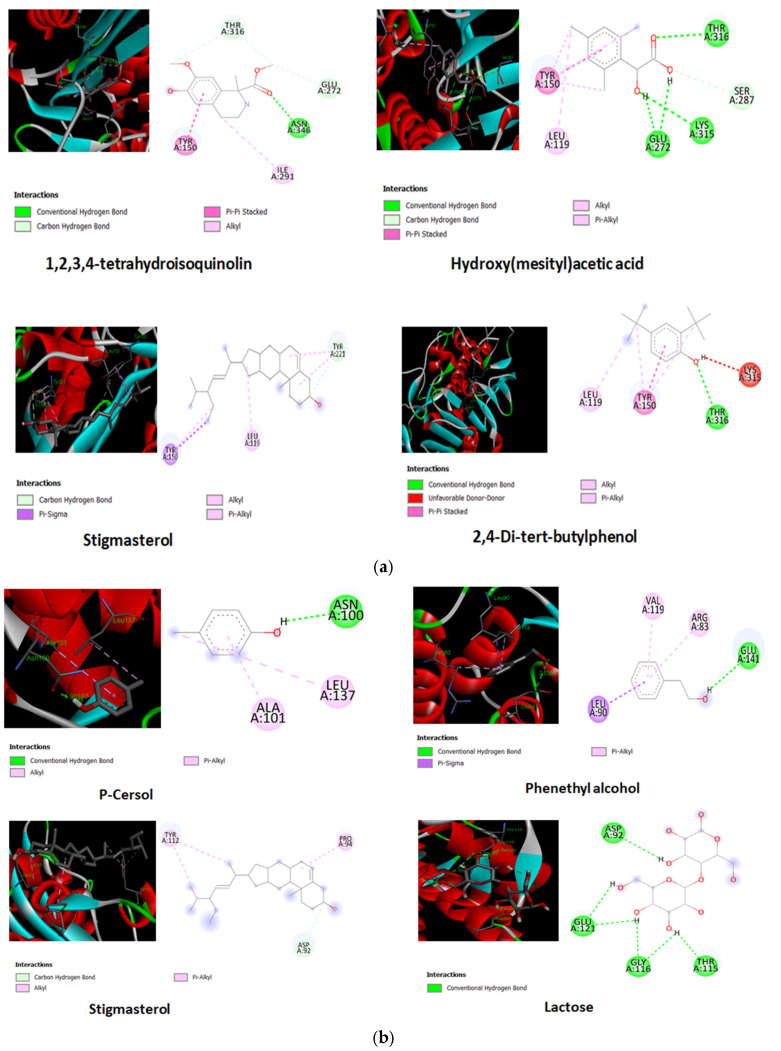
Molecular docking of the identified bioactive compound in F1 with (**a**) Ampc and (**b**) carbapenemases.

After GC-MS analysis, 19 bioactive compounds were identified from F1 and subjected to the AmpC and Carbapenemases macromolecules to determine their binding affinity (Table S1 and Figures S13–S20) and among 19 compounds, Stigmasterol, 1,2,3,4-tetrahydroisoquinolin, Lactose, Hydroxy(mesityl)acetic acid and 2,4-Di-tert-butylphenol exhibited good binding with the AmpC macromolecule as shown in Table 1. Compounds stigmasterol, lactose, phenethyl alcohol, and p-cresol exhibited good binding scores with carbapenemase macromolecules, as shown in Table 1. Compounds showed hydrogen bonding interactions with Tyr221, Asn346, Thr316, Ala292, Arg296, Tyr150, Glu272, Lys315, Ser287, Gly317, and Ala318 amino acid residues of the AmpC enzyme and hydrophobic interactions with Arg148, Tyr150, Leu119, Ile291, and Lys315 amino acid residues of the AmpC enzyme. Similarly, compounds showed Asp92, Glu121, Gly116, Thr115, Glu141, Asn100, Gly98, Tyr97, Lys140, Gln87, Pro94, Arg96 amino acid residues of the carbapenemases macromolecules and the Pro94, Tyr112, Arg83, Leu90, Val119, Leu137, Ala101, Arg96, Ile95, and Pro94 amino acid residues of the carbapenemases macromolecule as shown in Figure 6.

In molecular docking analyses, bioactive compounds showed binding interactions with amino acid residues of the selected macromolecules, including AmpC and Carbapenemases, which are similar to those reported in the literature [36,37,38]. These results suggest that the plant extract may be beneficial for the treatment of urinary tract infections.

#### 4.4.2. Density Functional Theory (DFT)

The absence of imaginary frequencies indicated the stability of the molecular geometry obtained. Further, the negative eigenvalues of HOMO and LUMO and the stability of the bioactive compounds were confirmed by the determination of positive ΔE values (Table 4). The energy gap between HOMO and LUMO (ΔE) is an indicator of significance toward charge transfer capability (Figure 7a,b). Notably, a decrease in ΔE corresponds with reduced excitation energies required for an electron to move from HOMO to LUMO, hence increasing charge transfer. The topology of frontier molecular orbitals showed that HOMO is distributed over substituted units on the thiaazolo-azepine core moiety, and LUMO is distributed mainly over the thiadiazolo-unit of the thiadiazolo-azepine moiety. This analysis suggested that substituted units over thiaazolo-azepine contributed to antioxidant properties. The ΔE values indicate that 3 and 4 have the smallest values of 5.705 eV and 5.75 eV, respectively, and thus have the softest molecules, with Enhanced chemical reactivity, increased polarizability and hyperpolarizability, and decreased kinetic stability.

Furthermore, the bioactive compound’s ability to donate electrons is enhanced by a lower IP and ΔE, which, in turn, results in higher antioxidant capacity [39]. Furthermore, higher antioxidant properties are associated with multiple other biological activities, such as antibacterial, anti-inflammatory, antiviral, and antidepressant activities. The obtained IP values of these compounds follow the order: 4 > 2 > 3 > 1. On the other hand, the order of antioxidant properties of these compounds, based on calculated energy gaps, is 3 > 2 > 4 > 1. Based on the cumulative effects of IP and ΔE, compound 3 will exhibit greater electron-donating capacity and related biological activities. This indicates that the antioxidant activity of the compounds is directly proportional to the number of electron-donating substituents.

Furthermore, the positive sign of χ and the negative sign of μ indicate that the bioactive compounds can accept electrons from the surrounding environment, thereby lowering their energy levels. The global electrophilicity index (ω) has been proposed to quantify the stabilization of molecular energy using Ƞ and χ. Reference [40] suggested that the electrophilic character of a molecule is a function of the square of its chemical potential, divided by its hardness. The electrophilicity index is the energy reduction resulting from the maximum electron transfer between a donor and an acceptor. A high electrophilicity index indicates greater bioactivity of the molecule. The calculation showed that compound 4 (ω = 2.068) has been found to show a higher electrophilic index (Table 4 and Figure 7b).

#### 4.4.3. Cytokine Profiling by ELISA

In the present study, IL-6 and IL-8 levels were compared across six groups: normal, diseased, standard herbal extract, standard antibiotic, low-dose fraction, and high-dose fraction. IL-6 is an important proinflammatory cytokine produced by mononuclear macrophages, fibroblasts, and endothelial cells and is involved in immune activation, B-cell differentiation, and acute-phase protein synthesis. Elevated IL-6 levels contribute to chronic inflammation, tissue damage, and immune dysregulation. IL-8, a highly efficacious chemokine produced by epithelial cells, mononuclear macrophages, and fibroblasts, is a key neutrophil chemoattractant that mediates immune cell recruitment and amplifies the inflammatory response to bacterial infections. Chronically elevated IL-8 levels, however, may promote excessive neutrophil recruitment, leading to tissue damage and fibrosis [41].

**Figure 8:**
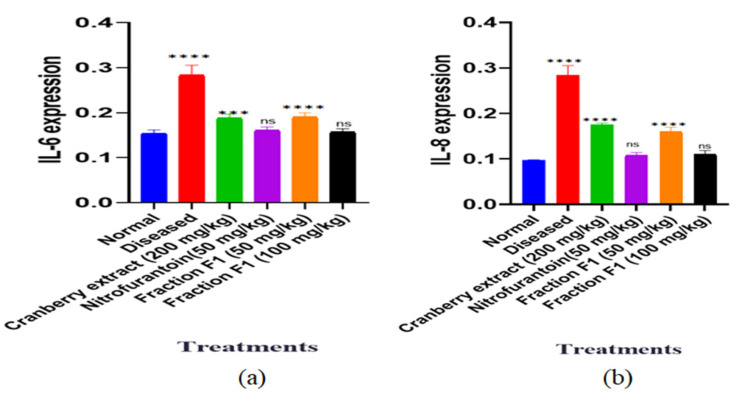
Effects of F1 on (**a**) IL-6 (**b**) IL-8. One-way ANOVA was conducted, and *** *p* < 0.001, **** *p* < 0.0001 was taken as statistically significant and ns as non-significant. The error bars presented are the standard error of the mean.

The diseased group showed elevated IL-6 and IL-8 levels compared with the normal group, reflecting a more pronounced inflammatory response associated with chronic urinary tract infection. Both the standard herbal extract and the standard antibiotic treatment reduced IL-6 and IL-8 levels compared with the diseased group. However, IL-6 and IL-8 levels were higher than in the normal group. Consequently, both the low-dose and high-dose fraction groups showed a dose-dependent reduction in IL-6 and IL-8, with the high-dose fraction group exhibiting the most significant reduction (Figure 8a,b). The findings indicate that all treatment groups reduced IL-6 and IL-8 levels, with the high-dose fraction demonstrating the most excellent anti-inflammatory effect. These results highlight the potential of the tested interventions—particularly the high-dose fraction—to reduce inflammation and modulate immune responses in chronic urinary tract infections.

#### 4.4.4. Gene Expression Analysis by RT-PCR

**Figure 9:**
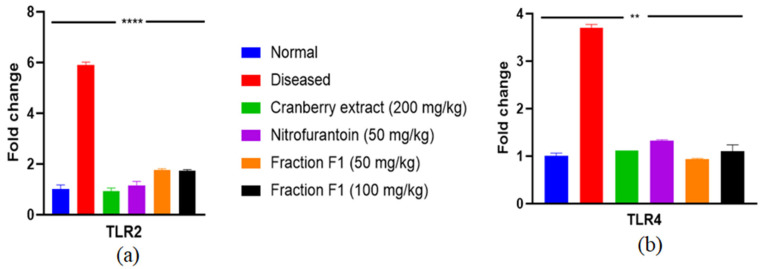
Effects of F1 on RNA expression of (**a**) TLR2 and (**b**) TLR4 in bladder tissue. Two-way ANOVA was conducted, and ** *p* < 0.004 and **** *p* < 0.0001 were taken as statistically significant. The error bars presented are the standard error of the mean.

To better comprehend how F1 regulates *Escherichia* spp. induced UTIs, we examined the expression of key genes, including TLR2 and TLR4 (Table 5), which are essential in UTI pathogenesis. The outcomes revealed that TLR2 and TLR4 expression in bladder tissues from normal individuals is lower than in the positive group, suggesting that elevated expression of these receptors may contribute to septic shock and inflammation. In comparison, the expression of these genes in Cranberry extract, Nitrofurantoin, and the low- and high-dose F1 groups was significantly higher than in the normal group but lower than in the positive group (Figure 9a,b). This showed that F1 could suppress TLR2 and TLR4 mRNA expression and reduce the overproduction of inflammatory mediators, thereby reducing the inflammatory response to UTIs in rats.

**Table 5:** The primer sequence and annealing temperature of the targeted genes.

Target Gene	Primer Sequence Forward	Primer Sequence Reverse	Annealing Temperature
TLR 2	5′ -TCTGAGTTCCGTGACATAGG-3′	3′ -AGATGTAACGCAACAGATTC-5′	59.1 °C
TLR 4	5′ -GTGAGCATTGATGAGTTCAG-3′	3′ -CATCTAATGATTGATAAGGATT-5′	59.1 °C

#### 4.4.5. Biochemical Studies

**Figure 10:**
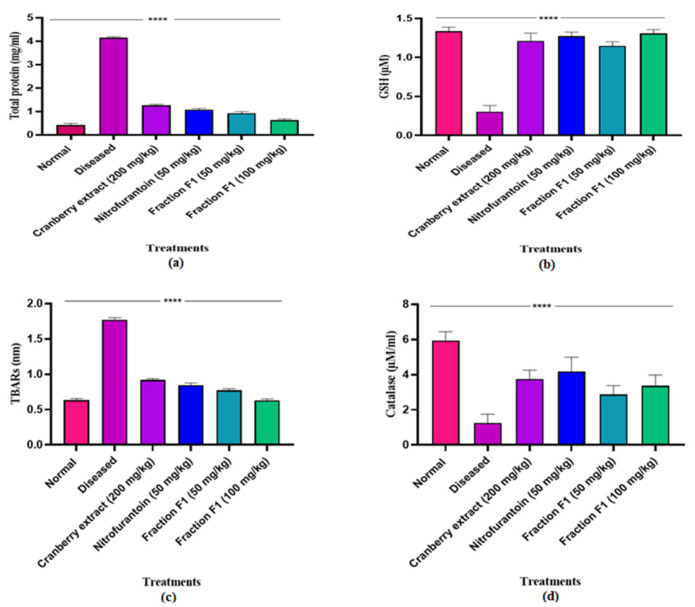
Effect of F1 on oxidative stress in bladder tissue (**a**) Total protein, (**b**) GSH, (**c**) Lipid peroxidase, (**d**) Catalase. One-way ANOVA was conducted, and **** *p* < 0.0001 was taken as statistically significant. The error bars presented are the standard error of the mean.

The fraction demonstrated significant protective effects against oxidative stress in infected bladder tissues by restoring key biochemical markers. Lipid peroxidation (TBARS) levels were notably elevated in infected tissues, indicating oxidative damage, but were markedly reduced following treatment (Figure 10c), suggesting membrane stabilization. Similarly, glutathione (GSH) levels, which were depleted due to infection-induced oxidative stress, showed significant restoration, highlighting improved antioxidant defense (Figure 10b). Catalase activity, which was initially suppressed in infected tissues, was effectively enhanced by the fraction, indicating improved enzymatic antioxidant response (Figure 10d). Additionally, the fraction stabilized total protein levels, preventing structural and functional protein degradation associated with oxidative damage (Figure 10a). These findings collectively suggest that the fraction mitigates oxidative stress and supports tissue recovery in urinary tract infections.

#### 4.4.6. Histopathology

**Figure 11:**
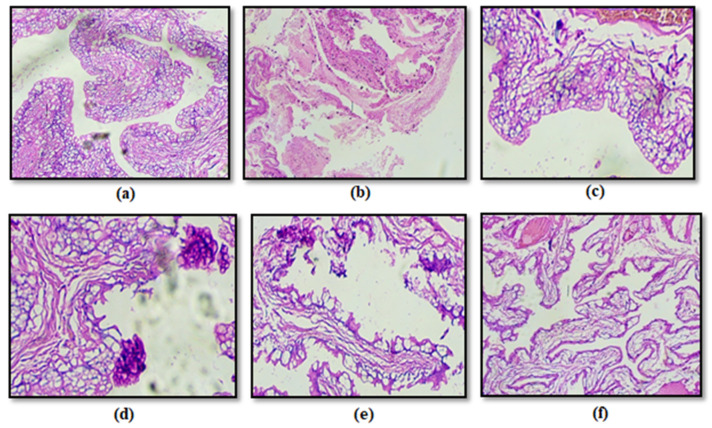
Histopathological study of the rat’s urinary bladder in *Escherichia* spp. induced UTI model. (**a**) Normal group showing normal histology, (**b**) Diseased group showing desquamation, (**c**) Cranberry extract (200 mg/kg) (herbal standard) and (**d**) Nitrofurantoin (50 mg/kg) (antibiotic standard) showed some desquamations and congestions, (**e**) F1 (50 mg/kg) (low dose of F1) group showed Desquamation, reactive hyperplasia of the urinary epithelium, dilation and congestion of bladder vessels with leukocyte infiltration and (**f**) F1 (100 mg/kg) (High dose of F1) showed nearly normal histology of urinary bladder.

The Histopathological evaluation of bladder tissue showed that the transitional epithelium, with its basement membrane, separates the epithelium from the adjacent lamina propria; in the control group, this structure was normal and intact (Figure 11a), indicating that the tissue structure remained unscathed. On the other hand, the diseased group exhibited gross structural damage to the bladder’s cellular framework, with severe tissue disruption due to infection (Figure 11b). The standard treatment groups (Figure 11c,d) showed desquamation with reactive hyperplasia of the urinary epithelium. In contrast, dilation and congestion of the blood vessels of the bladder with leukocyte infiltration were there, indicating inflammation and immune response in the fraction F1 (50 mg/kg) (low-dose) group also (Figure 11e). However, the fraction F1 (100 mg/kg) (high-dose) treatment group (Figure 11f) showed nearly normal bladder histology, indicating very minimal damage to the tissue, which suggests that this amount of dosing well restored the tissue to an almost healthy state.

## 5. Limitations

The current study provides strong preclinical evidence for the antibacterial and anti-inflammatory activities of the chloroform fraction (F1) of *C. callosus* leaf in the treatment of urinary tract infections; however, several challenges warrant in-depth investigation. The study employed a validated experimental model with *Escherichia* spp., a key uropathogen, to provide proof of concept. Involving a broader spectrum of clinical uropathogens in subsequent studies will enhance the translational value of the findings. Secondly, while in silico docking and DFT experiments helped provide mechanistic insight, isolating each bioactive and determining their contributions in vivo will provide an overall picture of the bioactive molecules. The efficacy and safety of F1 were established in rodent models under controlled conditions, and future work investigating pharmacokinetic profiling and dose optimization in more advanced experimental models will help enable clinical use. These challenges constitute a natural continuum in phytopharmaceutical development and do not detract from the novelty and quality of the current findings. Standardization of the chloroform fraction (F1) is critical because the phytochemical composition may vary with plant source, harvest season, geographic origin, and extraction method. Accordingly, future studies should define chemical fingerprints based on validated LC–MS–identified marker compounds and establish quantitative specifications to ensure batch-to-batch uniformity. Batch-to-batch variability is a common concern in natural products research, particularly for complex multi-component extracts, which require rigorous quality-control processes, reproducible extraction procedures, and comprehensive stability testing. Concerning regulation, any further development of F1 as a phytopharmaceutical would need to follow established regulatory frameworks, including safety, toxicology, pharmacokinetic profiling, and GMP considerations, as defined for botanical drug products by regulatory agencies worldwide. These comments are not a criticism of the study herein but rather constitute a logical extension toward clinical translation.

## 6. Discussion

The present study integrates phytochemical, computational, microbiological, and pharmacological approaches to evaluate the effectiveness of the chloroform fraction (F1) from leaves of *C. callosus* against *Escherichia* spp.-caused urinary tract infections. The fraction revealed potent antibacterial action with a minimum MIC of 2 μg/mL and MBC of 195 μg/mL, and hence holds promise to be used as an antimicrobial agent. Several in vitro assays have established that F1 induces destruction of bacterial membrane integrity (inner and outer), enhances permeability, inhibits metabolic enzymes such as dehydrogenases, and leads to leakage of cytoplasmic contents, collectively supporting a multifaceted bactericidal activity. LC-MS analysis identified various bioactive compounds, including steroids, phenolics, and alkaloids. Interestingly, some bioactive compounds—namely Stigmasterol and Hydroxy(mesityl)acetic acid—had remarkable binding affinities to AmpC and Carbapenemase enzymes as revealed by docking simulations. These enzymes are known to play essential roles in the establishment of β-lactam resistance, suggesting that the F1 fraction may counteract antimicrobial resistance (AMR) mechanisms. The in vivo results supplemented in vitro observations, showing a dose-dependent reduction in bacterial load and restoration of bladder morphology. Treatment with high-dose F1 (100 mg/kg) significantly reduced inflammation and tissue damage as observed in histopathology evaluation. Furthermore, Cytokine ELISA analysis showed a significant reduction in serum levels of the pro-inflammatory cytokines IL-6 and IL-8, indicating the systemic anti-inflammatory potential of F1 and its regulation of TLR-2 and TLR-4 gene expression, key mediators of inflammation induced by bacterial infection. The downregulation of these genes indicates reduced inflammation, which in turn may be attributed to the efficient elimination of bacteria following F1 treatment. Furthermore, F1 treatment groups exhibited favorable redox profiles, as evidenced by increased catalase and GSH levels and reduced TBARS, indicating robust antioxidant defenses against infection-induced oxidative stress. Based on the in vitro, in silico, and in vivo results, we propose that the active fraction F1 first disrupts bacterial membrane integrity, as evidenced by increased membrane permeability and protein leakage, thereby facilitating the intracellular entry of bioactive phytocompounds. The internalized phytocompounds then inhibit key bacterial enzymes, including AmpC and carbapenemases, as evidenced by molecular docking, which was further corroborated by a dehydrogenase inhibition assay, resulting in impaired metabolic activity and reduced cell viability. This was accompanied by modulation of oxidative stress, with reduced lipid peroxidation and restored antioxidant enzyme levels, thereby further disrupting bacterial homeostasis. Suppression of TLR2 and TLR4 signaling and of pro-inflammatory cytokines (IL-6 and IL-8) during in vivo supplementation complemented these antibacterial effects by alleviating host inflammatory damage and promoting tissue recovery. Collectively, these interlinked events culminate in the inhibition of bacterial growth, clearance of infection, and restoration of bladder tissue integrity, representing a coherent anti-bacterial and therapeutic mechanism. Collectively, these findings indicate that F1 exhibits broad-spectrum therapeutic activity against UTIs by exerting antibacterial, antioxidant, and anti-inflammatory effects.

## 7. Conclusions

This study provides strong experimental evidence supporting the traditional use of *C. callosus* in the treatment of urinary tract infections. The chloroform fraction (F1) exhibited strong antibacterial activity against *Escherichia* spp. because of the disruption of bacterial membrane integrity, inhibition of metabolic enzymes, and suppression of oxidative and inflammatory responses. LC-MS and molecular docking studies identified several bioactive compounds with high affinities for prominent resistance-associated bacterial enzymes and potential roles in counteracting antimicrobial resistance. The in vivo model confirmed a significant reduction in bacterial burden, inflammation, and oxidative stress, along with increased histological protection of bladder tissue. These findings place the *C. callosus* fraction F1 as a potential candidate for phytotherapeutic development in the management of urinary tract infections, thereby providing a rationale for further clinical research.

## 8. Future Perspective

To enhance the therapeutic potential of *C. callosus* in the treatment of UTI, future research should isolate and characterize the individual bioactive components responsible for these effects. Their respective pharmacological targets and mechanisms of action should be unraveled using omics-based and receptor–ligand interaction studies. Pharmacokinetic and pharmacodynamic studies, including bioavailability, metabolism, tissue distribution, and long-term safety, will be of utmost importance for clinical use. Assessing the efficacy of F1 in polymicrobial infection models and its combination with conventional antibiotics may provide insights into synergistic approaches to treating drug-resistant UTIs. Finally, progression to phase I clinical trials will be required to determine the safety and therapeutic efficacy of *C. callosus*-derived products in human subjects. Formulating drugs into appropriate dosage forms, such as capsules, syrups, or intravesical preparations, may improve patient adherence and therapeutic efficacy in clinical practice.

## Figures and Tables

**Table 1:** MIC and MBC of extracts of *C. callosus*.

S. No.	Parameters	CCAq	CCM	CCHM	Standard
1	MIC (μg/mL)	10	5	5	5
2	MBC (μg/mL)	350	350	300	350

**Table 2:** MIC, MBC of Fractions of *C. callosus*.

S. No.	Parameters	F1	F2	F3	Standard
1	MIC (μg/mL)	2	4	20	5
2	MBC (μg/mL)	195	200	200	200

**Table 3:** Major bioactive compounds of F1.

S. No.	Compound Name	Retention Time	Molecular Formula	Molecular Weight	Compound Nature
1.	p-Cresol	11.902	C7 H8 O	108.0572	Phenolic compound
2.	Phenylethyl Alcohol	13.182	C8 H10 O	122.0727	Aromatic compound
3.	4H-Pyran-4-one, 2,3-dihydro-3,5-dihydroxy -6-methyl	2.806	C6 H8 O4	144.0418	Flavonoid fraction
4.	5-Hydroxymethylfurfural	9.956	C6 H6 O3	126.0314	Aldehyde compound
5.	Benzeneethanol, 4-hydroxy-	15.527	C8 H10 O2	138.0677	Phenolic compound
6.	Benzene, 1-(1,5-dimethyl-4-hexenyl) -4-methyl	16.541	C15 H22	202.1713	Aromatic compound
7.	Lactose	2.191	C12 H22 O11	342.1162	Sugar moiety
8.	Dodecanoic acid	20.016	C12 H24 O2	200.1795	Lauric acid
9.	Tetradecanoic acid	21.978	C14 H28 O2	228.2104	Myristic acid
10.	Hexadecanoic acid, methyl ester	28.181	C17 H34 O2	270.2571	Palmitic acid ester
11.	Palmitoleic acid	25.720	C16 H30 O2	254.2264	Palmitoleic acid
12.	n-Hexadecanoic acid	19.484	C16 H32 O2	256.2394	Palmitic acid
13.	9,12-Octadecadienoic acid (Z,Z)	26.136	C18 H32 O2	280.2392	Linoleic acid
14.	Oleic Acid	29.245	C18 H34 O2	282.2548	Oleic Acid
15.	Octadecanoic acid	22.195	C18 H36 O2	284.2706	Stearic acid
16.	3,4-Dihydroisoquinoline, 1-[3-methoxybenzyl] -6-methoxy	14.978	C18 H19 N O2	281.1412	Alkaloid
17.	4HDibenzo[de,g] quinoline,5,6,6a,7- tetrahydro-10,11- dimethoxy-6-methyl-, (R)-	20.948	C19 H21 N O2	295.1565	Alkaloid
18.	(-)-1,2,3,4- Tetrahydroisoquinolin-6-ol-1- carboxylic acid, 7-methoxy-1-methyl-, methyl ester	7.213	C13 H17 N O4	251.1154	carboxylic acid
19.	Stigmasterol	24.772	C29 H48 O	412.3704	Steroid
20.	New compound	21.064	C14 H22 O	206.1688	Phenolic compound
21.	Hydroxy(mesityl)acetic acid (2,4,6-Trimethylmandelic acid)	22.727	C11 H14 O3	194.0937	aromatic carboxylic acid

**Table 4:** Different quantum reactivity descriptors, such as energy of HOMO, LUMO, energy gap, ionization potential, electron affinity, molecular softness, electronegativity, and electrophilic index of the title bioactive compounds.

	HOMO (E_H_; eV)	LUMO (E_L_; eV)	E Gap (ΔE; eV)	Ionization Potential (I, eV)	Electron Affinity (A, eV)	Electronegativity (χ; eV)	Chemical Potential (μ; eV)	Chemical Hardness (η; eV)	Chemical Softness (*σ*)	Electrophilicity Index (*ω*)
1	−6.377	0.283	6.66	6.377	−0.283	3.047	−3.047	3.33	0.15	1.394
2	−6.013	−0.263	5.75	6.013	0.263	3.138	−3.138	2.875	0.174	1.713
3	−5.955	−0.25	5.705	5.955	0.25	3.103	−3.103	2.853	0.175	1.687
4	−6.551	−0.515	6.036	6.551	0.515	3.533	−3.533	3.018	0.166	2.068

## Data Availability

The original contributions presented in this study are included in the article/Supplementary Material. Further inquiries can be directed to the corresponding authors.

## Supplementary Materials

The following supporting information can be downloaded at: https://www.mdpi.com/article/10.3390/pathogens15010111/s1, Figure S1: Total ion chromatogram of all isolated bioactive constituents; Figure S2: LC-MS chromatogram of (a) p-Cresol (b) Phenylethyl Alcohol; Figure S3: LC-MS chromatogram of (a) 4H-Pyran-4-one, 2,3-dihydro-3,5-dihydroxy-6-methyl (b) 5-Hydroxymethylfurfural; Figure S4: LC-MS chromatogram of (a) Benzeneethanol, 4-hydroxy- (b) Benzene, 1-(1,5-dimethyl-4-hexenyl)-4-methyl; Figure S5: LC-MS chromatogram of (a) Lactose (b) Dodecanoic acid; Figure S6: LC-MS chromatogram of (a) Tetradecanoic acid (b) Hexadecanoic acid, methyl ester; Figure S7: LC-MS chromatogram of (a) Palmitoleic acid (b) n-Hexadecanoic acid; Figure S8: LC-MS chromatogram of (a) 9,12-Octadecadienoic acid (Z,Z), (b) Oleic Acid; Figure S9: LC-MS chromatogram of (a) Octadecanoic acid (b) 3,4-Dihydroisoquinoline, 1-[3-methoxybenzyl]-6-methoxymethyl; Figure S10: LC-MS chromatogram of (a) 4HDibenzo[de,g]quinoline,5,6,6a,7-tetrahydro-10,11-dimethoxy-6-methyl-, (R)- (b) (-)-1,2,3,4 Tetrahydroisoquinolin-6-ol-1-carboxylic acid, 7-methoxy-1-methyl-, methyl ester; Figure S11: LC-MS chromatogram of (a) Stigmasterol (b) New compound; Figure S12: LC-MS chromatogram of Hydroxy(mesityl)acetic acid (2,4,6-Trimethylmandelic acid); Figure S13: 2D and 3D poses of Ligand-receptor complex of 4H-Pyran-4-one, 2,3-dihydro-; 5 Hydroxymethylfurfural; 9,12-Octadecadienoic acid; Benzeneethanol, 4-hydroxy- with AmpC; Figure S14: 2D and 3D poses of Ligand-receptor complex of Dodecanoic acid; Hexadecanoicacid, methyl; Lactose; n-Hexadecanoic acid with AmpC; Figure S15: 2D and 3D poses of Ligand-receptor complex of Octadecanoic acid; Oleic Acid; Palmitoleic acid; P-CERSOL with AmpC; Figure S16: 2D and 3D poses of Ligand-receptor complex of Phenethyl alcohol; Tetradecanoic acid with AmpC; Figure S17: 2D and 3D poses of Ligand-receptor complex of 1,2,3,4-tetrahydroisoquinolin; 4H-Pyran-4-one, 2,3-dihydro-; 5-Hydroxymethylfurfural; 9,12-Octadecadienoic acid with Carbapenemases; Figure S18: 2D and 3D poses of Ligand-receptor complex of Benzeneethanol, 4-hydroxy-; Dodecanoic acid; Hexadecanoic acid, methyl; Hydroxy(mesityl)acetic acid with Carbapenemases; Figure S19: 2D and 3D poses of Ligand-receptor complex of n-Hexadecanoic acid; Octadecanoic acid; Oleic acid; Palmitoleic acid with Carbapenemases; Figure S20: 2D and 3D poses of Ligand-receptor complex of Tetradecanoic acid; 2,4-Di-tert-butylphenol with Carbapenemases; Table S1: Molecular docking studies of identified test compounds from plant fraction with AmpC and Carbapenemases.

## Abbreviations

The following abbreviations are used in this manuscript:

ANOVA	One-way Analysis of Variance
*C. callosus*	*Cucumis callosus*
cDNA	Complementary DNA
DFT	Density Functional Theory
DNA	Deoxyribonucleic Acid
ELISA	Enzyme-Linked Immunosorbent Assay
FE-SEM	Field Emission Scanning Electron Microscopy
HOMO	Highest Occupied Molecular Orbital
LC-MS	Liquid Chromatography–Mass Spectrometry
LUMO	Lowest Unoccupied Molecular Orbital
MBC	Minimum Bactericidal Concentration
MIC	Minimum Inhibitory Concentration
MTT	3-(4,5-Dimethylthiazol-2-yl)-2,5-Diphenyltetrazolium Bromide
OD	Optical Density
PBS	Phosphate-Buffered Saline
PCR	Polymerase Chain Reaction
qRT-PCR/RT-PCR	Quantitative Real-Time PCR
ROS	Reactive Oxygen Species
SD	Standard Deviation
TLR	Toll-Like Receptor
TLR-2	Toll-Like Receptor 2
TLR-4	Toll-Like Receptor 4
Two-way ANOVA	Two-way Analysis of Variance
UTI	Urinary Tract Infection
WBCs	White Blood Cells
