# Supplementary material for: Natural Protective Mechanisms of Cucumis callosus Leaves in Escherichia Species-Induced Urinary Tract Infection: An Integrated In Silico and In Vivo Study
Source: Pathogens. 2026 Jan 19;15(1):111. doi: 10.3390/pathogens15010111 (PMC12845193; doi:10.3390/pathogens15010111)
Supplement: Supplementary file 1 [file pathogens-15-00111-s001.zip › pathogens-4063109-supplementary.pdf]

Natural Protective Mechanisms of *Cucumis callosus* Leaves in *Escherichia species*-Induced Urinary Tract Infection: An Integrated in Silico and In Vivo Study

Liquid chromatography with tandem mass spectrometry

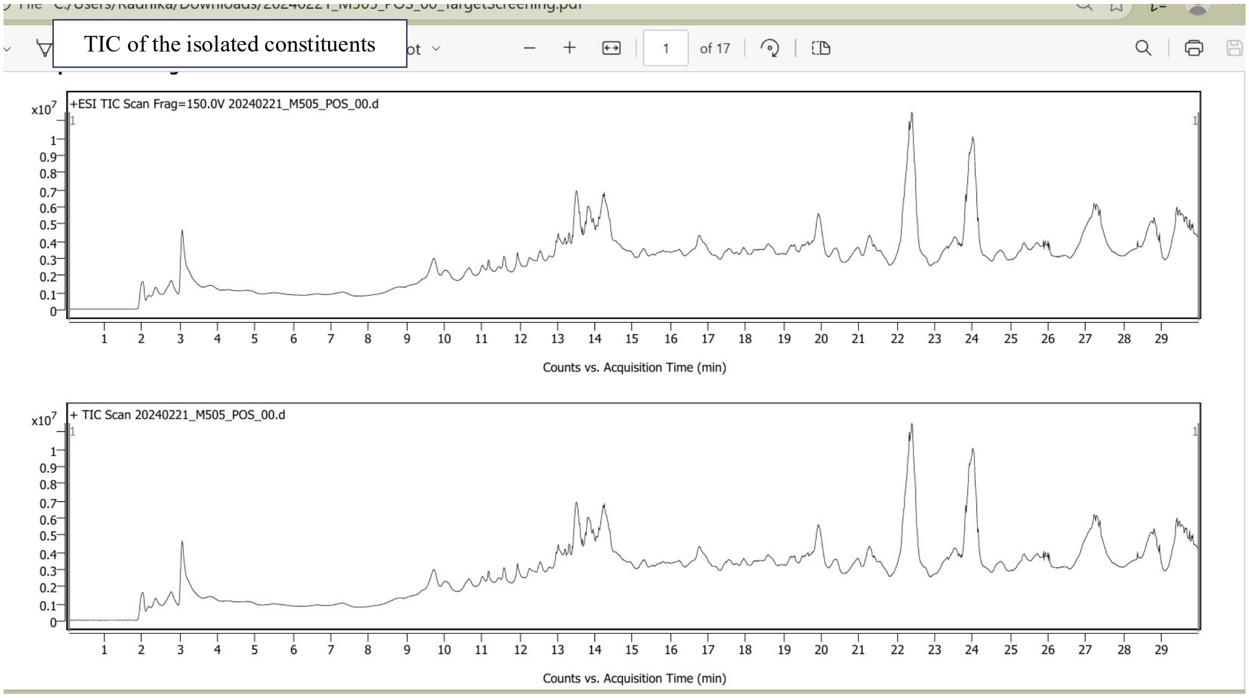

Figure S1: Total ion chromatogram of all isolated bioactive constituents

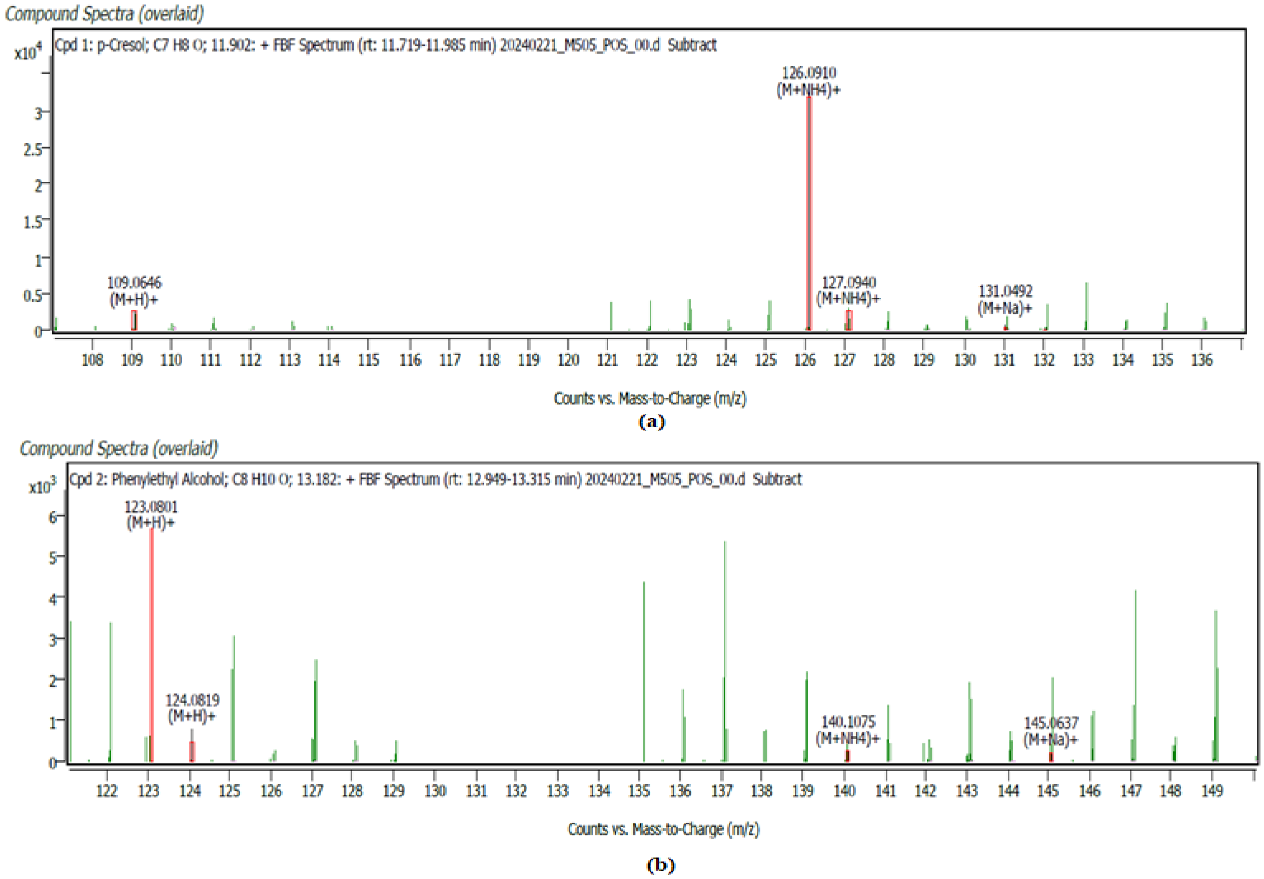

Figure S2: LC-MS chromatogram of (a) p-Cresol (b) Phenylethyl Alcohol

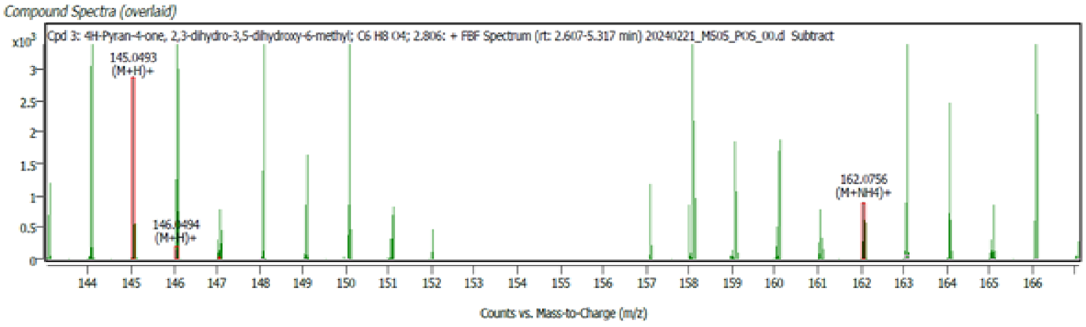

(a)

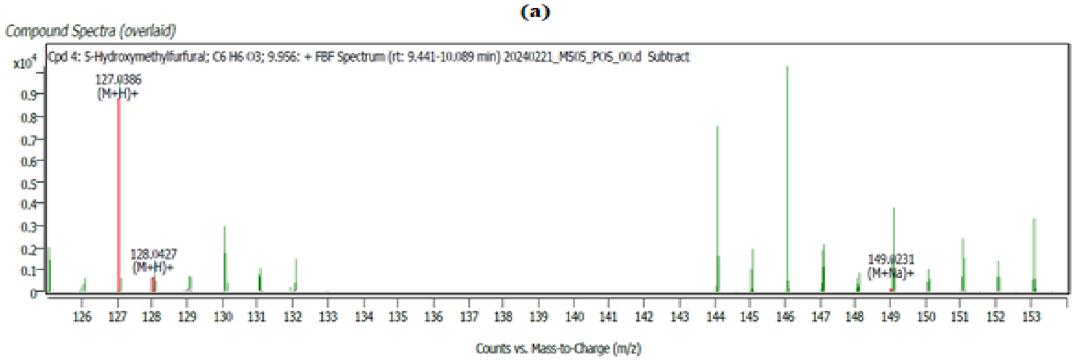

(b)

Figure S3: LC-MS chromatogram of (a) 4H-Pyran-4-one, 2,3-dihydro-3,5-dihydroxy-6-methyl (b) 5-Hydroxymethylfurfural

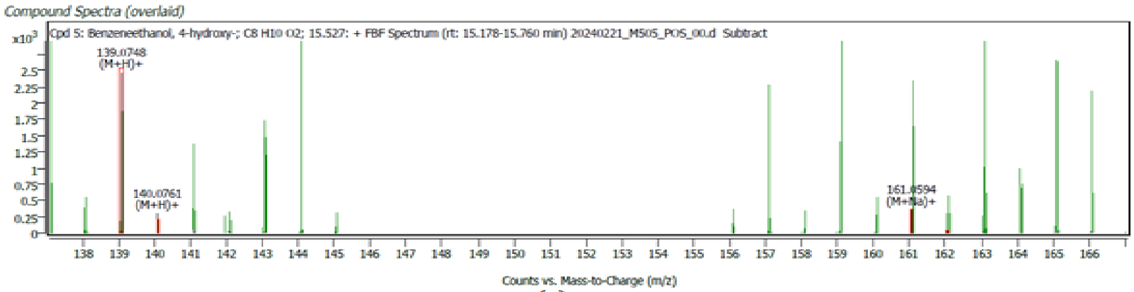

(a)

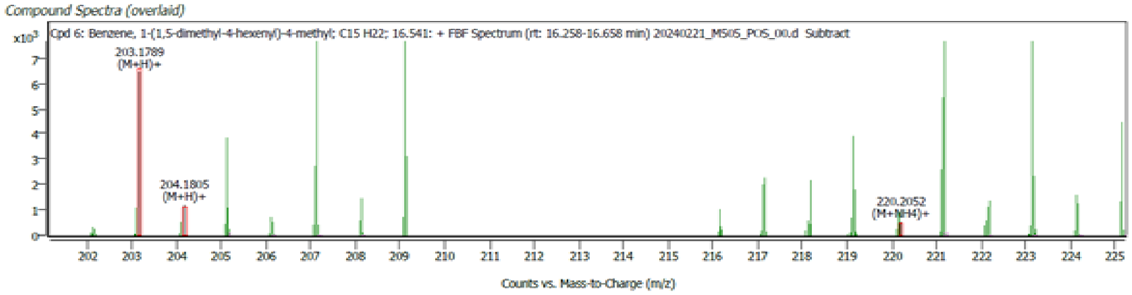

(b)

Figure S4: LC-MS chromatogram of (a) Benzeneethanol, 4-hydroxy- (b) Benzene, 1-(1,5-dimethyl-4-hexenyl)-4-methyl

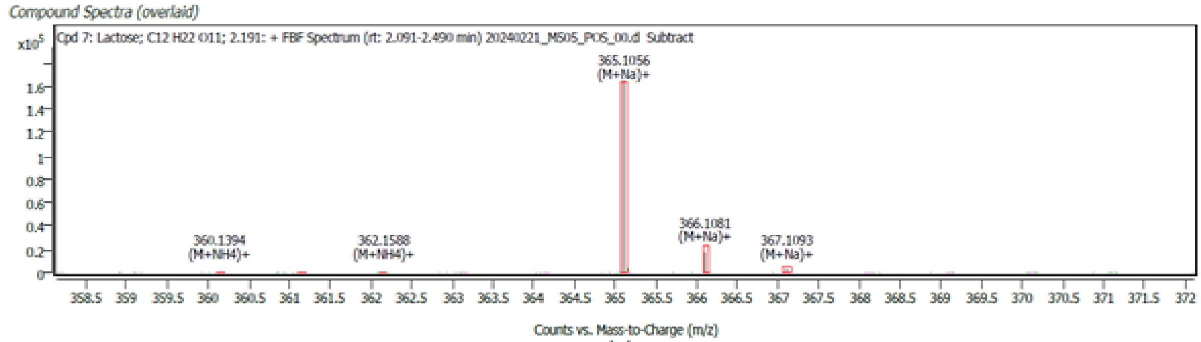

(a)

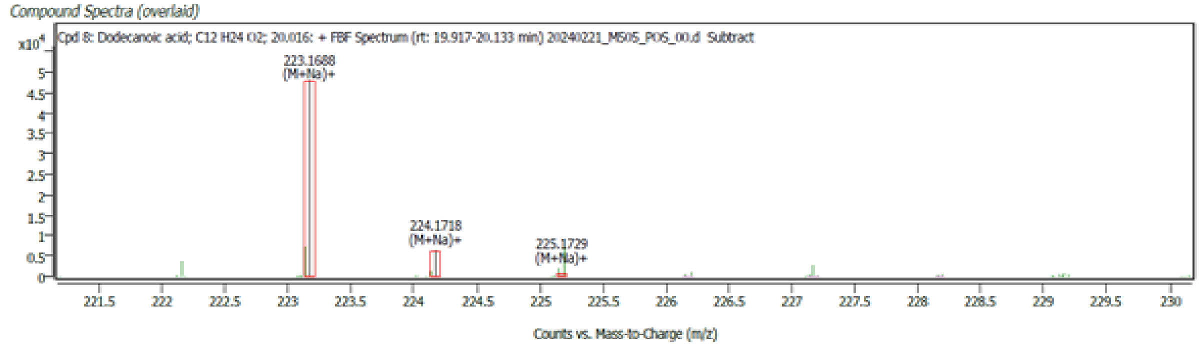

(b)

Figure S5: LC-MS chromatogram of (a) Lactose (b) Dodecanoic acid

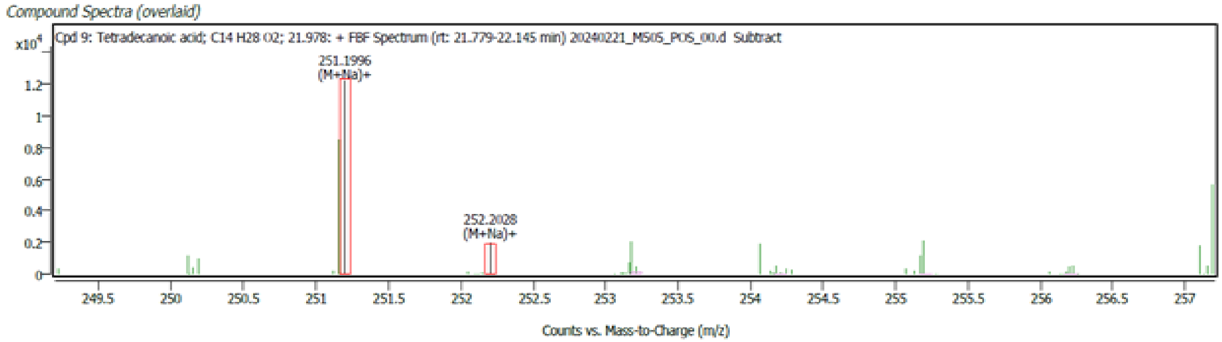

(a)

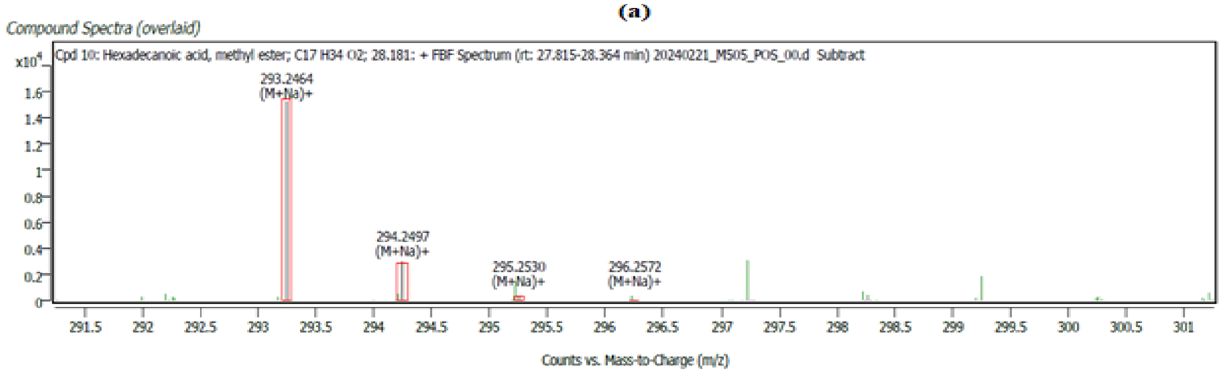

(b)

Figure S6: LC-MS chromatogram of (a) Tetradecanoic acid (b) Hexadecanoic acid, methyl ester

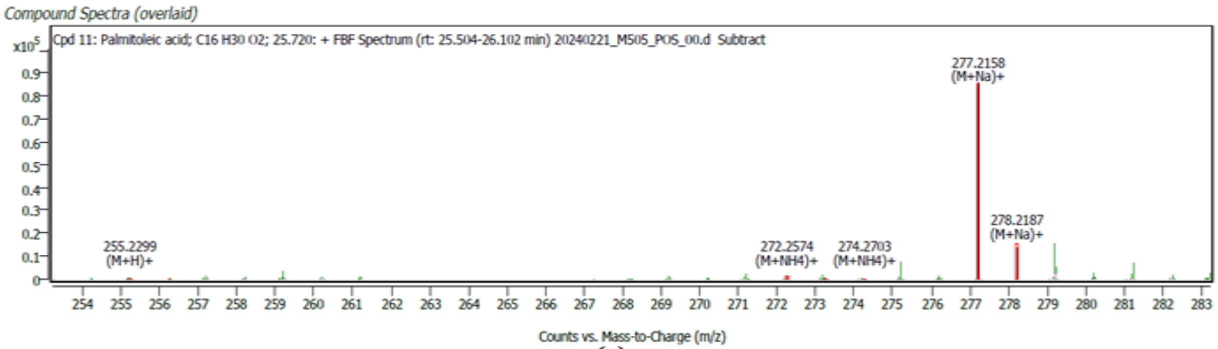

(a)

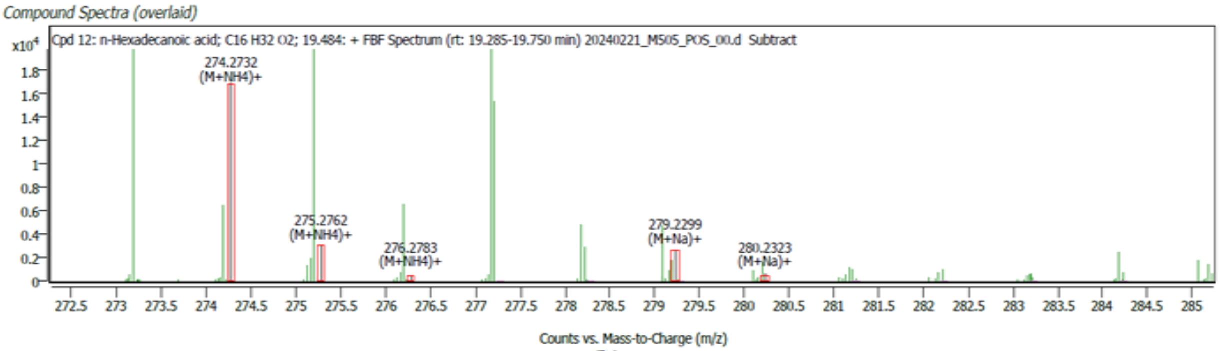

(b)

Figure S7: LC-MS chromatogram of (a) Palmitoleic acid (b) n-Hexadecanoic acid

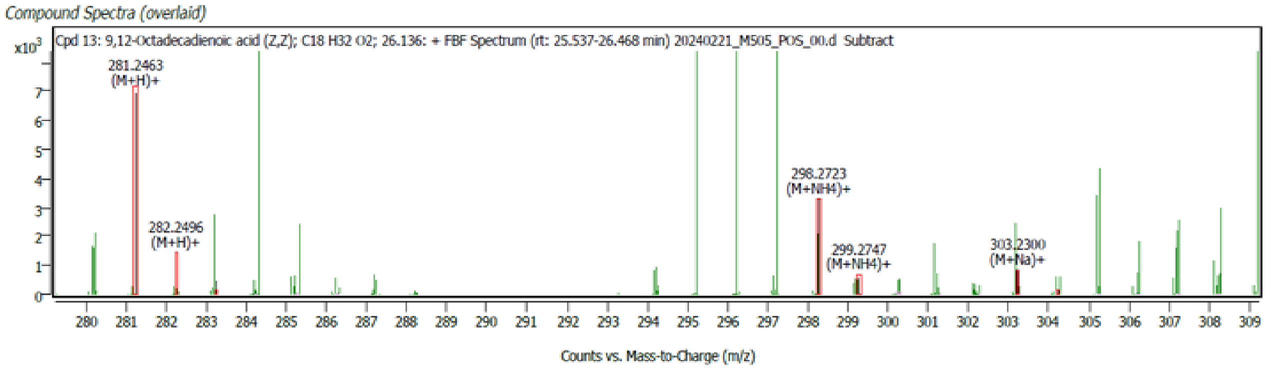

(a)

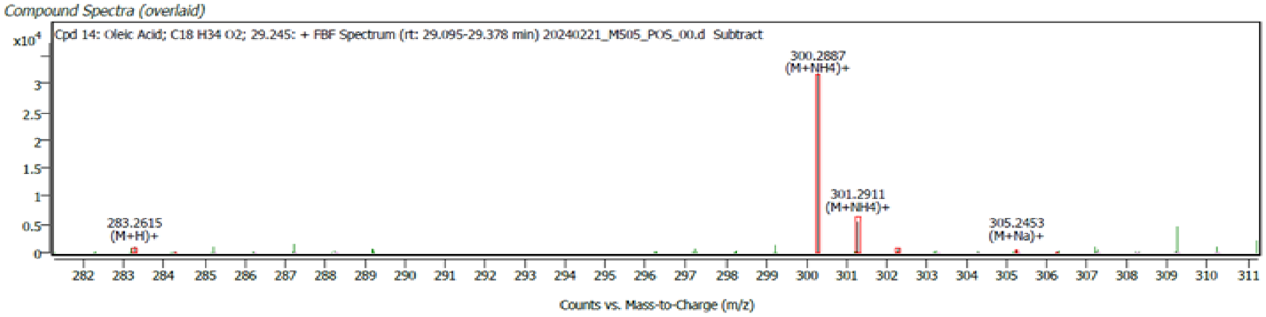

(b)

Figure S8: LC-MS chromatogram of (a) 9,12-Octadecadienoic acid (Z,Z), (b) Oleic Acid

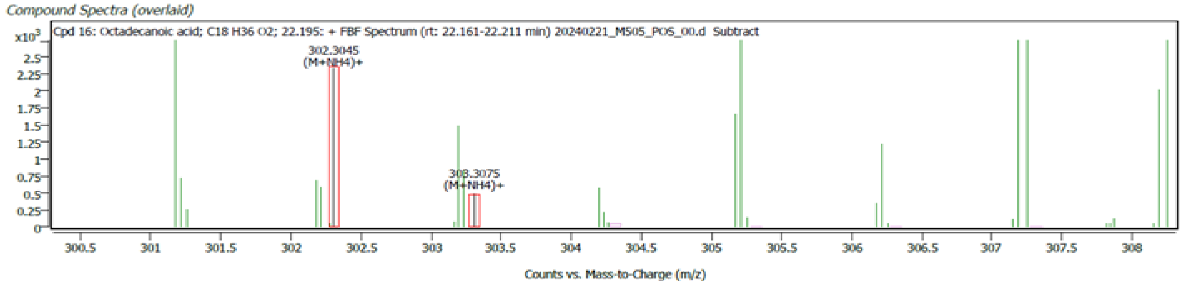

(a)

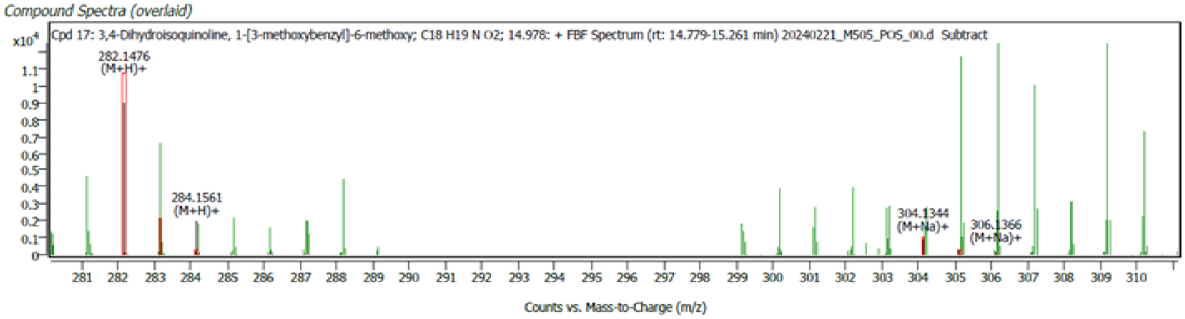

(b)

Figure S9: LC-MS chromatogram of (a) Octadecanoic acid (b) 3,4-Dihydroisoquinoline, 1-

[3-methoxybenzyl]-6-methoxymethyl

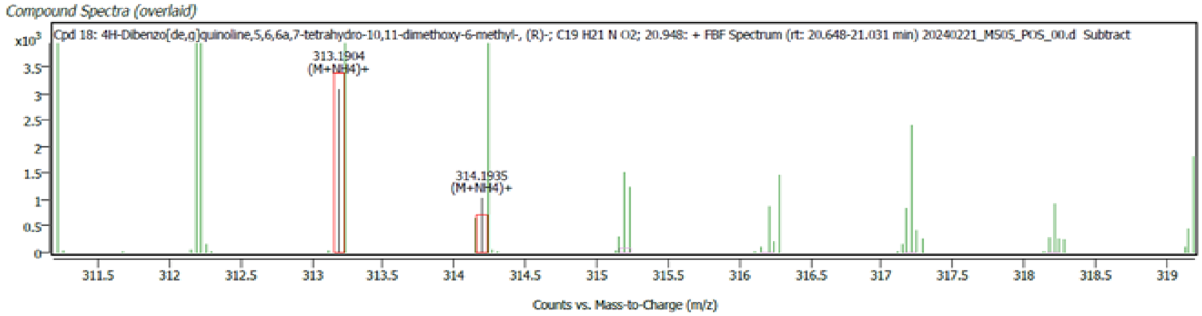

(a)

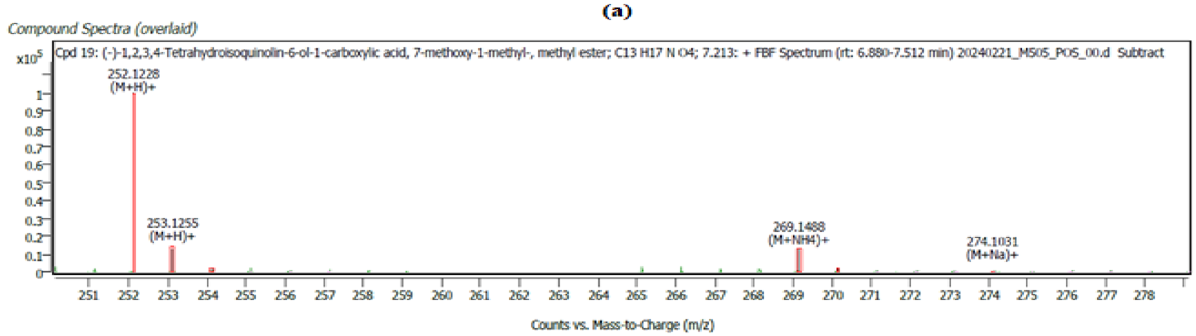

(b)

Figure S10: LC-MS chromatogram of (a) 4HDibenzo[de,g]quinoline,5,6,6a,7-tetrahydro-10,11-dimethoxy-6-methyl-, (R)- (b) (-)-1,2,3,4 Tetrahydroisoquinolin-6-ol-1-carboxylic acid, 7-methoxy-1-methyl-, methyl ester

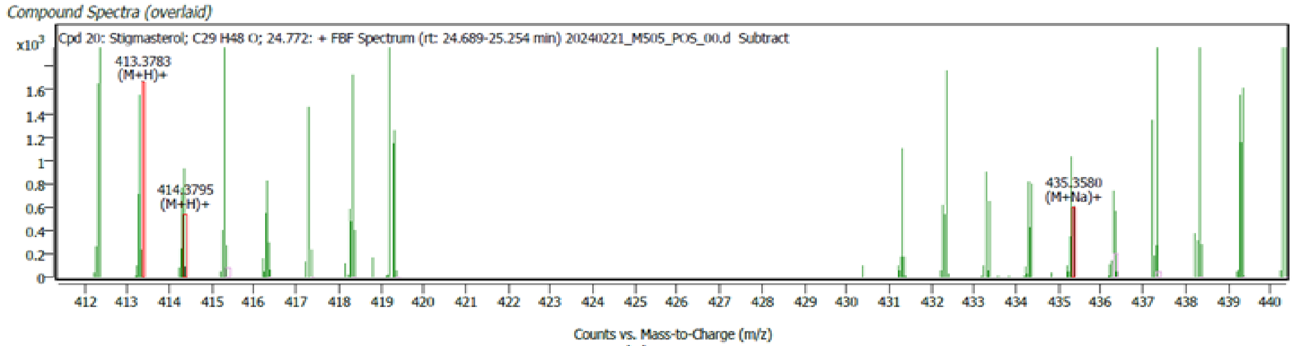

(a)

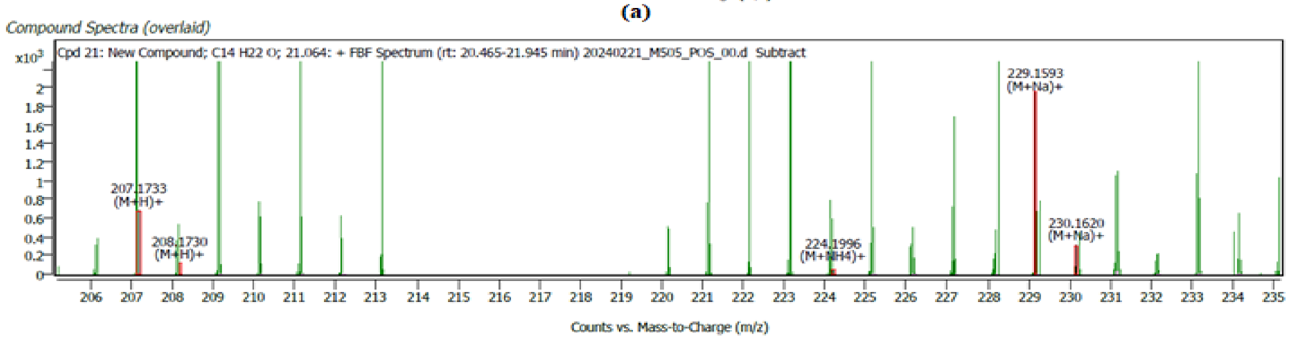

(b)

Figure S11: LC-MS chromatogram of (a) Stigmasterol (b) New compound,

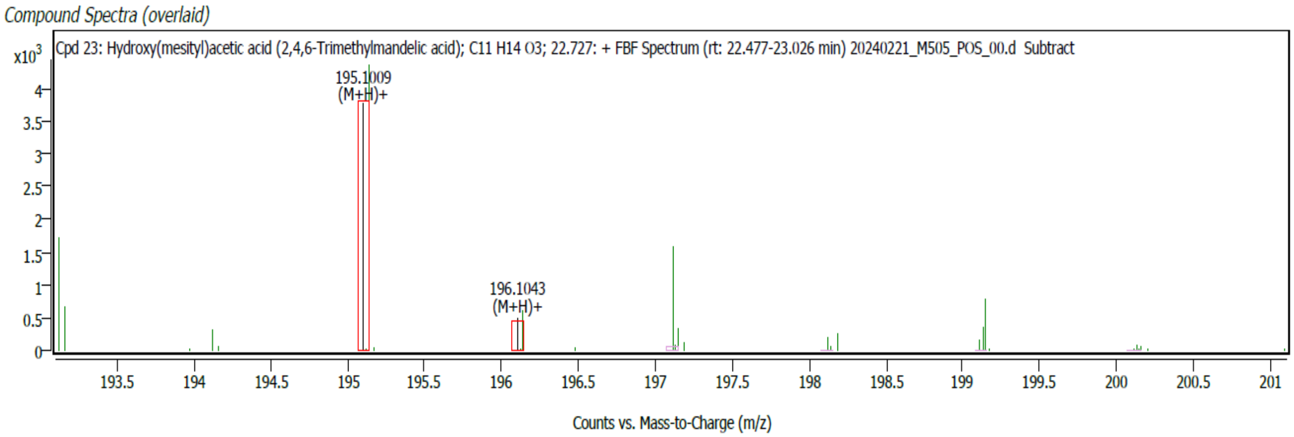

Figure S12: LC-MS chromatogram of Hydroxy(mesityl)acetic acid (2,4,6-Trimethylmandelic acid)

Molecular Docking

Table: S1 Molecular docking studies of identified test compounds from plant fraction with AmpC and Carbapenemases

S. No.	Compounds	Docking score		Bonding residues			
		AmpC	Carapen emases	AmpC (1iel)		Carbapenemases (6mey)	
				Hydrogen bond residues (Å)	Hydrophob ic residues (Å)	Hydrogen bond residues (Å)	Hydrophobic residues (Å)
**1.**	Lactose	87.6	64.5	H-Asn346 (2.885), O-Thr316 (3.025), O-Thr316 (3.018), H-Ala292 (3.045), O-Arg296 (3.363)	Arg148	H-Glu121 (2.581), H-Asp92 (2.420), H-Gly116 (2.635), H-Gly116 (2.360), H-Thr115 (2.085)	
**2.**	1,2,3,4-tetrahydroiso quinolin	88.8	57	O-Asn346 (3.250), C-Glu272 (3.570), C-Thr316 (3.335), C-Thr316 (3.795)	Tyr150, Ile291	H-Glu141 (2.521), N-Tyr97 (3.799)	Arg96, Ala101, Leu137
**3.**	n-Hexadecanoic acid	68.4	51	H-Asn346 (2.171), O-Thr316 (2.885), O-Gly317 (3.246)	Ile291, Leu119, Tyr150	H-Glu121 (2.616), H-Gly116 (2.299)	Pro94, Ile95, Arg96

**4.**	Octadecanoic acid	67.2	43.5	O-Thr316 (3.056), O-Ala318 (3.117)	Tyr150, Leu119	H-Asp92 (2.148), O-Thr118 (3.011)	Pro94, Tyr112
**5.**	Oleic acid	70.8	45	H-Asn346 (2.641), O-Thr316 (3.015), O-Thr316 (2.954)	Leu293, Leu119, Tyr150	H-Pro94 (2.560), O-Arg96 (2.927)	Ile95, Leu137, Ala101
**6.**	Palmitoleic acid	69.6	43.5	O-Thr316 (3.030), O-Ser64 (3.121)	Tyr150, Leu119	H-Tyr112 (3.279)	Pro94
**7.**	P-CERSOL	63.6	60	H-Glu272 (3.043)	Tyr150	H-Asn100 (1.903)	Leu137, Ala101
**8.**	Phenethyl alcohol	62.4	64.5	H-Thr316 (2.101), H-Thr316 (2.604), O-Tyr150 (3.240)	Tyr150	H-Glu141 (2.538)	Arg83, Leu90, Val119
**9.**	Stigmasterol	126	70.5	C-Tyr221 (3.270)		C-Asp92 (3.478)	Pro94, Tyr112
**10.**	Tetradecanoic acid	66	51	O-Arg296 (3.150), O-Arg148 (2.980), H-Glu272 (2.538)	Tyr150, Leu293, Leu119	O-Gln87 (3.201)	Pro94, Ile95, Arg96
**11.**	4H-Pyran-4-one, 2,3-dihydro-	64.8	54	O-Lys315 (3.022), O-Thr316 (3.019)	Tyr150	O-Lys140 (2.970), O-Gly98 (3.044)	

**12.**	5-Hydroxymethylfurfural	61.2	46.5	O-Thr316 (3.069), O-Thr316 (2.841), H-Thr316 (2.896), H-Lys315 (3.014)	Tyr150	O-Lys140 (3.186), O-Lys140 (3.243), H-Asn100 (2.135)	
**13.**	9,12-Octadecadienoic acid	70.8	52.5	O-Thr316 (2.996), O-Thr316 (2.908), H-Thr316 (2.804), H-Tyr150 (2.794)	Tyr150, Leu119, Lys315	O-Gln87 (2.988), H-Glu141 (2.320)	Ile95, Pro94, Arg96
**14.**	Benzeneethanol, 4-hydroxy-	67.2	51	H-Ile291 (2.697), O-Ala294 (3.277), H-Glu272 (1.949)	Tyr150	O-Gly98 (3.169), H-Asn100 (2.577)	Ala101
**15.**	Dodecanoic acid	61.2	42	O-Tyr150 (3.135), O-Lys315 (2.803), O-Thr316 (3.151), H-Thr316 (2.661)	Tyr150, Leu119	H-Asn100 (2.327), O-Asn100 (3.187)	Ile95
**16.**	Hexadecanoic acid, methyl	62.4	45	O-Ala318 (2.818), O-Ser64 (2.868)	Leu119, Tyr150	O-Thr115 (2.963)	Pro94, Tyr112
**17.**	Hydroxy(mesityl)acetic acid	84	58.5	H-Glu272 (2.232), H-Glu272 (2.272), O-Lys315 (3.169), O-Thr316 (3.017), O-Ser287 (3.492)	Tyr150, Leu119	O-Gly98 (3.052)	Ala101, Leu137, Lys140

**18.**	2,4-Di-tert-butylphenol	70.69	30.89	O-Thr316 (2.806)	Lys315, Leu119, Tyr150, Lys315	H-Arg96 (2.179)	Ile95

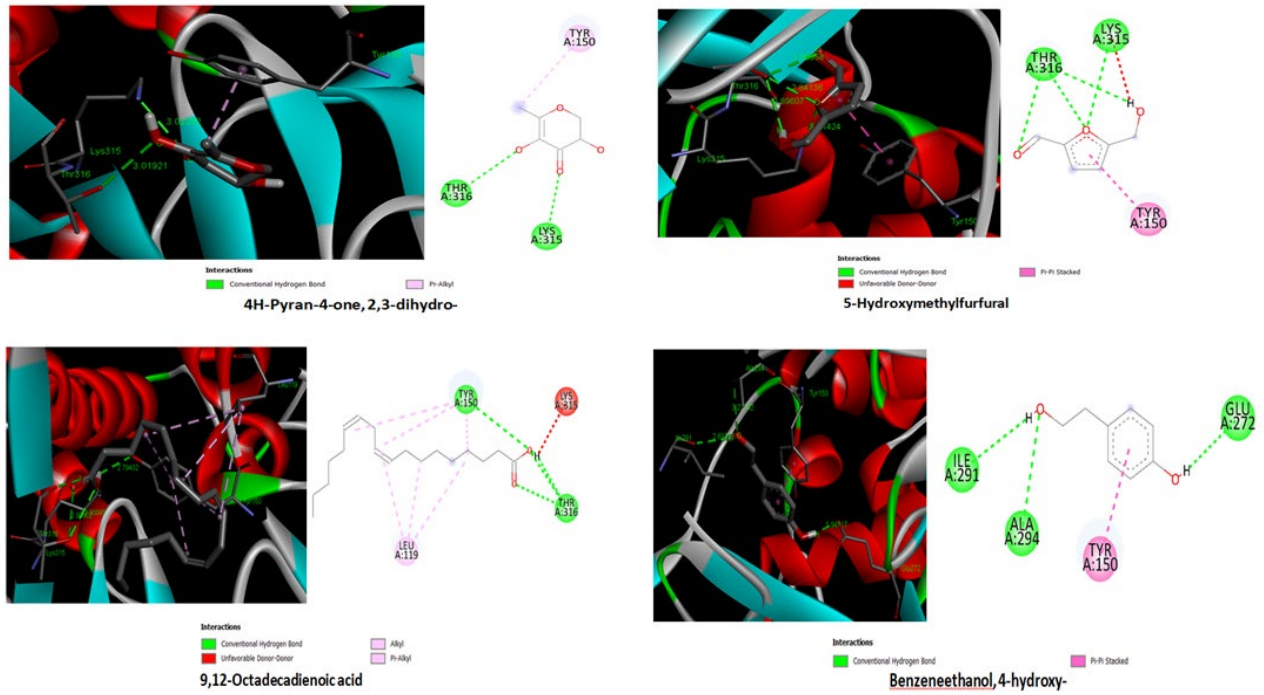

Fig S13: 2D and 3D poses of Ligand-receptor complex of 4H-Pyran-4-one, 2,3-dihydro-; 5 Hydroxymethylfurfural; 9,12-Octadecadienoic acid; Benzeneethanol, 4-hydroxy- with

AmpC

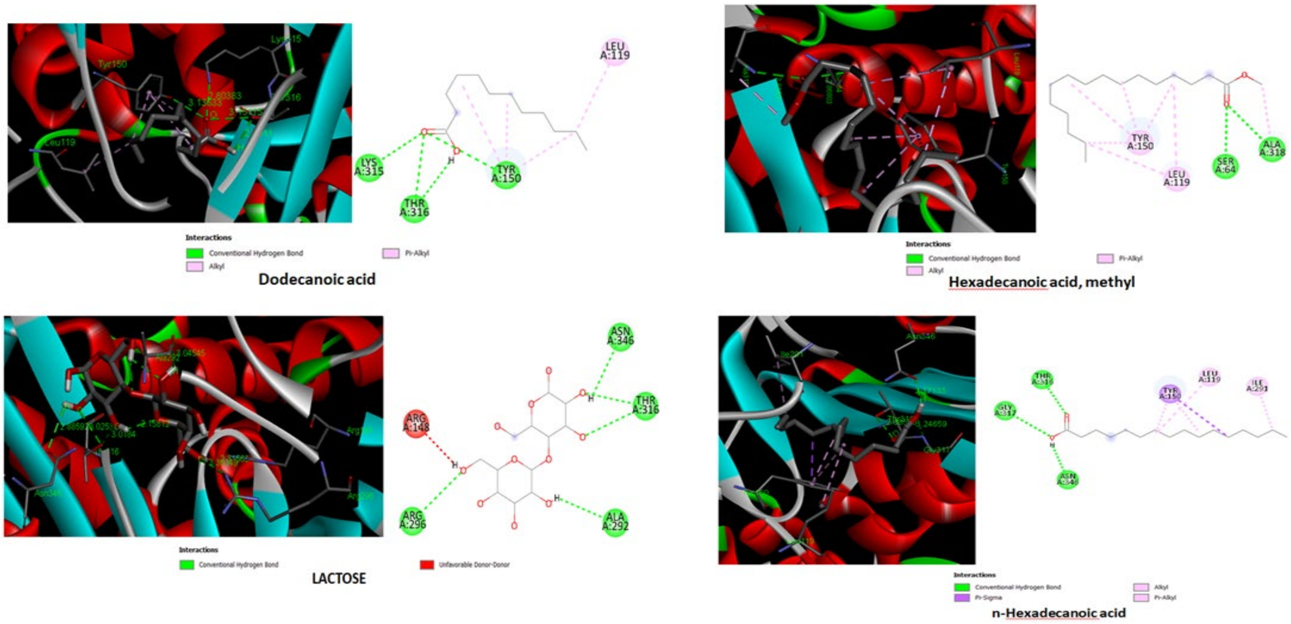

Fig S14: 2D and 3D poses of Ligand-receptor complex of Dodecanoic acid; Hexadecanoicacid, methyl; Lactose; n-Hexadecanoic acid with AmpC

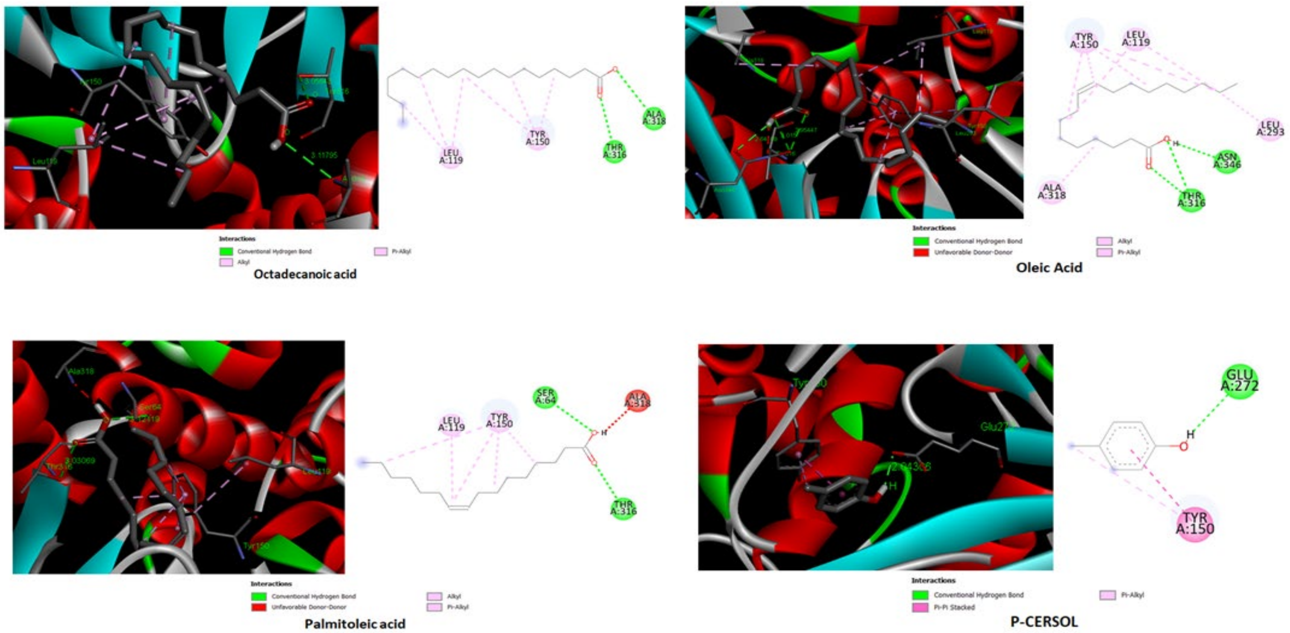

Fig S15: 2D and 3D poses of Ligand-receptor complex of Octadecanoic acid; Oleic Acid

; Palmitoleic acid; P-CERSOL with AmpC

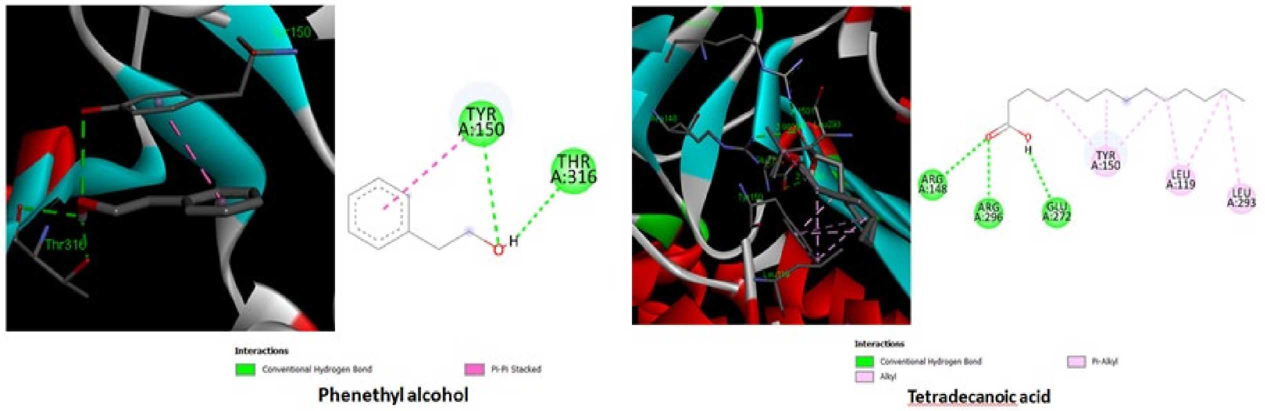

Fig S16: 2D and 3D poses of Ligand-receptor complex of Phenethyl alcohol; Tetradecanoic acid with AmpC

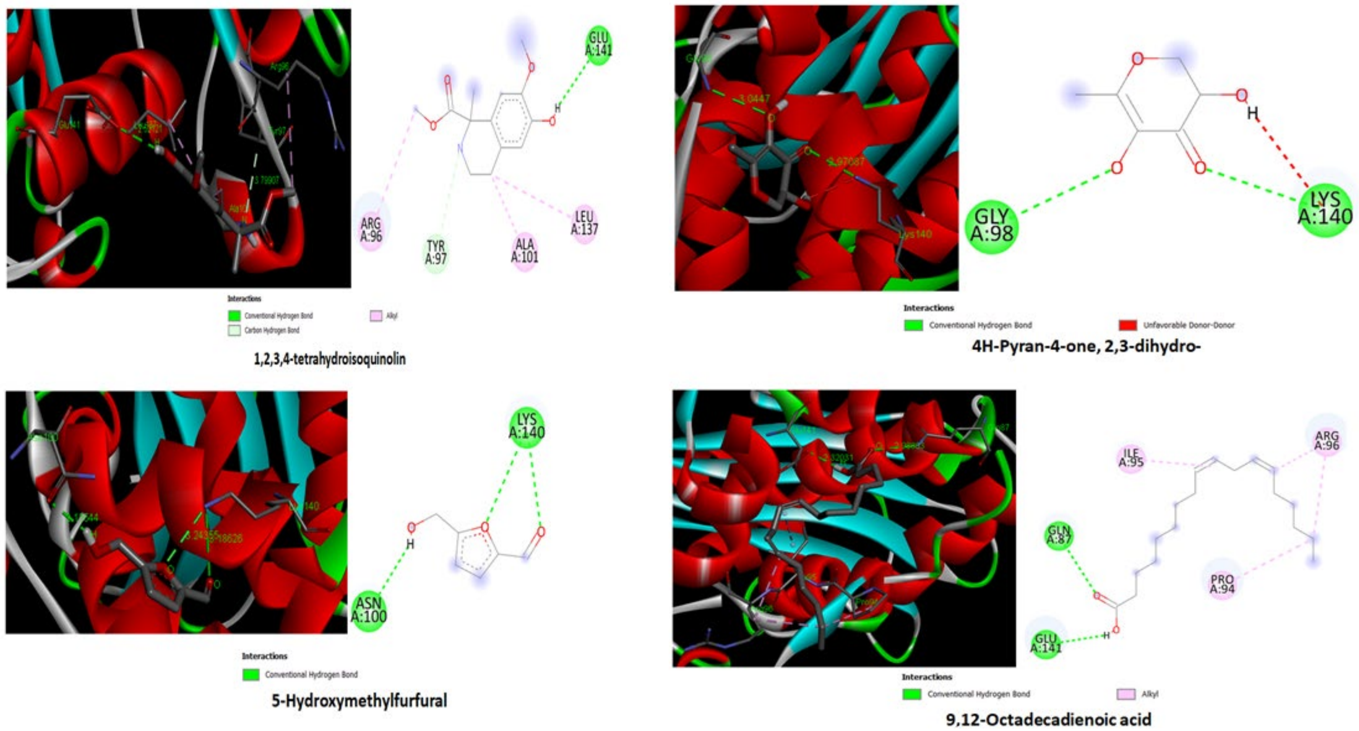

Fig S17: 2D and 3D poses of Ligand-receptor complex of 1,2,3,4-tetrahydroisoquinolin; 4H-Pyran-4-one, 2,3-dihydro-; 5-Hydroxymethylfurfural; 9,12-Octadecadienoic acid with Carbapenemases

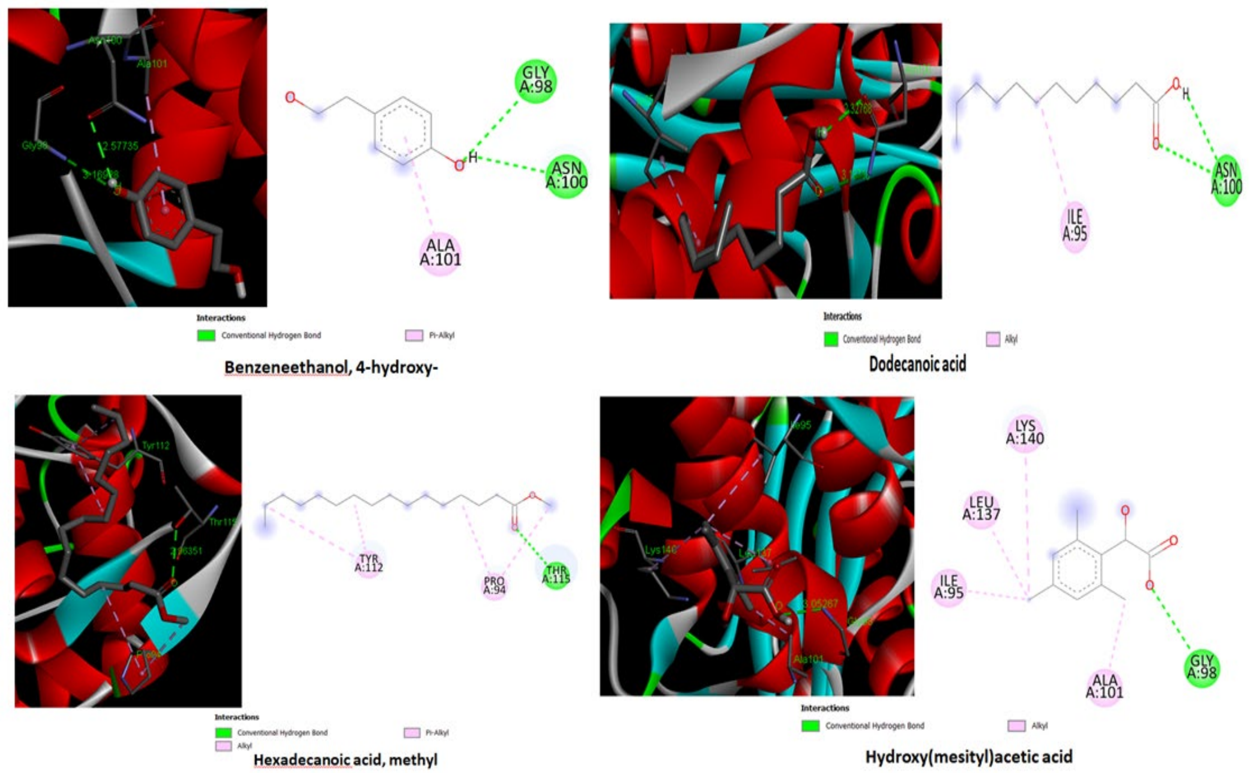

Fig S18: 2D and 3D poses of Ligand-receptor complex of Benzeneethanol, 4-hydroxy-; Dodecanoic acid; Hexadecanoic acid, methyl; Hydroxy(mesityl)acetic acid with Carbapenemases

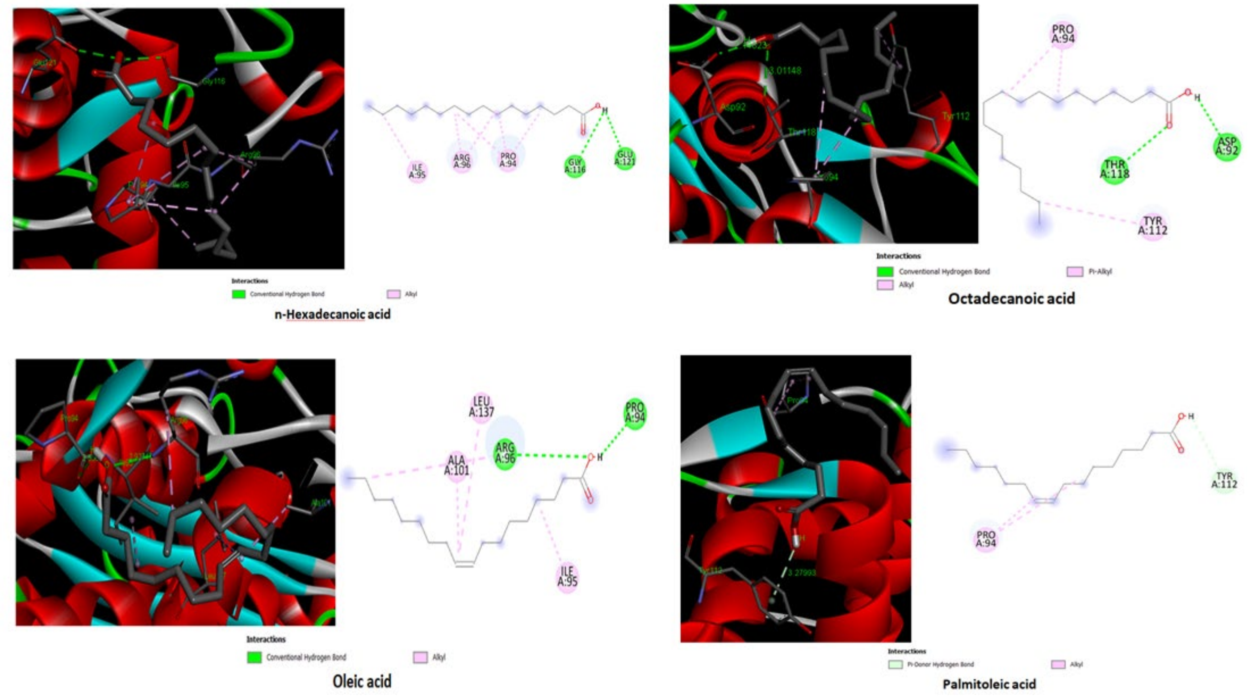

Fig S19: 2D and 3D poses of Ligand-receptor complex of n-Hexadecanoic acid; Octadecanoic acid; Oleic acid; Palmitoleic acid with Carbapenemases

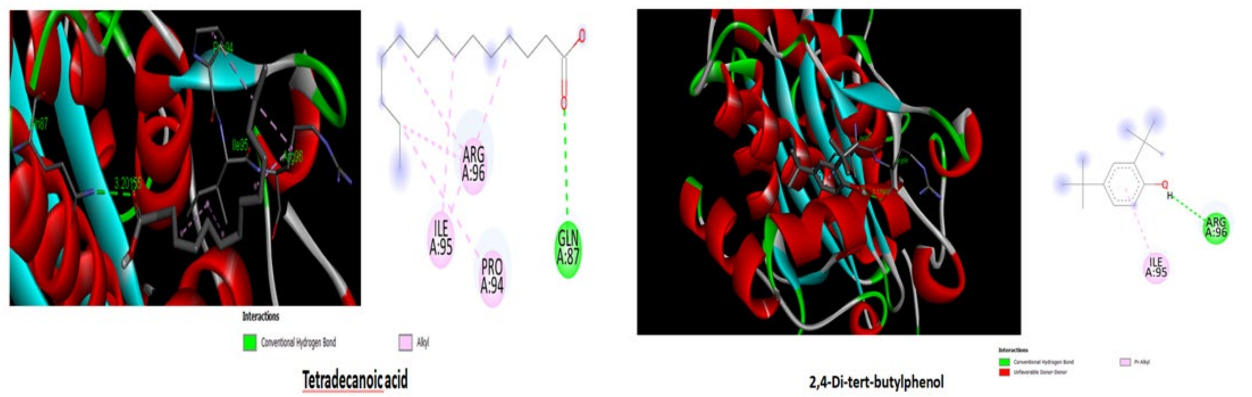

Fig S20: 2D and 3D poses of Ligand-receptor complex of Tetradecanoic acid; 2,4-Di-tert-butylphenol with Carbapenemases
